# Multi-factor data normalization enables the detection of copy number aberrations in amplicon sequencing data

**DOI:** 10.1093/bioinformatics/btu436

**Published:** 2014-07-12

**Authors:** Valentina Boeva, Tatiana Popova, Maxime Lienard, Sebastien Toffoli, Maud Kamal, Christophe Le Tourneau, David Gentien, Nicolas Servant, Pierre Gestraud, Thomas Rio Frio, Philippe Hupé, Emmanuel Barillot, Jean-François Laes

**Affiliations:** ^1^Inserm, U900, Bioinformatics, Biostatistics, Epidemiology and Computational Systems Biology of Cancer, ^2^Institut Curie, Centre de Recherche, 26 rue d’Ulm, Paris 75248, ^3^Mines ParisTech, Fontainebleau 77300, ^4^Inserm, U830, Genetics and Biology of Cancers, Paris 75248, France, ^5^Institut de Pathologie et de Génétique, Gosselies 6041, Belgium, ^6^Clinical Research Department, ^7^Department of Medical Oncology, ^8^Plateforme de Génomique, Département de recherche translationnelle, Centre de recherche, ^9^Next-generation sequencing platform, Institut Curie, ^10^CNRS, UMR144, Subcellular Structure and cellular Dynamics, Paris 75248, France and ^11^OncoDNA, Gosselies 6041, Belgium

## Abstract

**Motivation:** Because of its low cost, amplicon sequencing, also known as ultra-deep targeted sequencing, is now becoming widely used in oncology for detection of actionable mutations, i.e. mutations influencing cell sensitivity to targeted therapies. Amplicon sequencing is based on the polymerase chain reaction amplification of the regions of interest, a process that considerably distorts the information on copy numbers initially present in the tumor DNA. Therefore, additional experiments such as single nucleotide polymorphism (SNP) or comparative genomic hybridization (CGH) arrays often complement amplicon sequencing in clinics to identify copy number status of genes whose amplification or deletion has direct consequences on the efficacy of a particular cancer treatment. So far, there has been no proven method to extract the information on gene copy number aberrations based solely on amplicon sequencing.

**Results:** Here we present ONCOCNV, a method that includes a multifactor normalization and annotation technique enabling the detection of large copy number changes from amplicon sequencing data. We validated our approach on high and low amplicon density datasets and demonstrated that ONCOCNV can achieve a precision comparable with that of array CGH techniques in detecting copy number aberrations. Thus, ONCOCNV applied on amplicon sequencing data would make the use of additional array CGH or SNP array experiments unnecessary.

**Availability and implementation:** http://oncocnv.curie.fr/

**Contact:** valentina.boeva@curie.fr

**Supplementary information:** Supplementary data are available at *Bioinformatics* online.

## 1 INTRODUCTION

The emergence of the amplicon sequencing technique, which followed whole-exome sequencing (WES), promises a revolution in cancer diagnostics and treatment. Amplicon sequencing consists of the polymerase chain reaction (PCR) amplification of a limited number of the genomic regions of interest (amplicons) followed by high-throughput sequencing (Supplementary Fig. S1A) (Beadling *et al.*, 2013). Each amplicon often coincides with an exon; exons longer than the typical length of PCR reaction products may be covered by two or more amplicons (Supplementary Fig. S1B).

Although relatively expensive exome sequencing consists of in-depth sequencing of nearly all the coding exons, the amplicon sequencing technique aims at sequencing a limited number of genes (from several dozen to several thousand exons) at an extremely low cost. The genes included in a panel of amplicon sequencing (actionable genes) are genes that are often altered in different cancer types, and for whose alterations targeted therapies have been established or are in clinical development. For instance, the TargetRich™ CRX kit from Kailos Genetics assays such cancer-related genes as *BRAF*, *EGFR*, *FLT3*, *JAK2*, *KIT*, *KRAS*, *PIK3CA*, *PTEN*, *TP53* and *VEGFA*; the AmpliSeq™ Cancer Panel from Life Technologies targets 190 regions of interest in 46 well-characterized oncogenes and tumor suppressors.

Some actionable genes often undergo point mutation or exon deletions (e.g. *ALK, BRAF*), whereas others undergo amplification in copy number (e.g. *MYCN*, *ERBB2*) (Garraway and Lander, 2013; Small *et al.*, 1987). Because of the exceedingly high read coverage of amplicon sequencing data, there is no methodological issue in the identification of point mutations and small insertions or deletions (indels). However, how to reliably detect copy number changes, in particular gene deletions and amplifications, in amplicon sequencing data is still open to discussion.

Here, we focus on the identification of copy number alterations (CNAs) also known as large copy number changes (CNVs) of the actionable genes targeted by amplicon sequencing. Although there are several algorithms to detect CNAs in exome sequencing data (Amarasinghe *et al.*, 2013; Boeva *et al.*, 2011; Li *et al.*, 2012), it is questionable as to whether the same approaches can be efficient when applied to amplicon sequencing data. First, amplicon sequencing targets fewer regions and thus provides less information than exome sequencing datasets (<10 000 exons versus >200 000 exons); consequently, data normalization can be less effective on amplicon sequencing data. Second, because of the different protocols used for library preparation, amplicon sequencing data can have various biases. Importantly, while for exome sequencing experiments an effort has been made to uniform exon coverage, amplicon sequencing technology emphasizes extremely high depth of coverage with less regard to coverage homogeneity.

Here we provide a solution to the challenging question of extracting CNAs from amplicon sequencing data by (i) defining a method to normalize read coverage with a small set of normal control samples and (ii) assigning statistical significance to putative CNAs resulting from the segmentation of normalized profiles. We validated the proposed method on (A) a high amplicon density dataset of eight tumor samples for which array comparative genomic hybridization (array CGH) profiles were available, (B) a high amplicon density dataset of 30 ErbB2-positive ovarian cancer samples and (C) a low amplicon density dataset of 30 tumors, coupled with single nucleotide polymorphism (SNP) array data. We show that the results obtained from the ONCOCNV method compare favorably with the results obtained from ADTEx (Amarasinghe *et al.*, 2013) and NextGENe (http://www.softgenetics.com/NextGENe_013.html), which are respectively public-domain and commercial software designed to detect CNVs in whole exome sequencing data.

## 2 MATERIALS AND METHODS

**Fig. 1.**
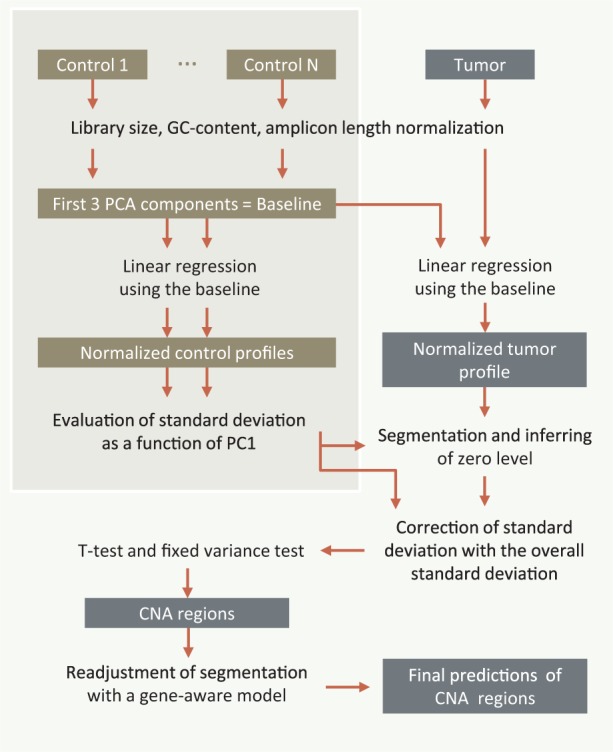
Workflow of ONCOCNV. Control samples may be processed separately from tumor samples. As ONCOCNV performs normalization by GC content and amplicon length, it is not critical for the control samples to be sequenced jointly

In this article, we present a method for detecting copy number changes in sequencing data generated for a relatively small panel of genes. We first apply several normalization steps: library-specific (normalization for library size, GC content and amplicon length) and technology-specific normalization (normalization with a control baseline); the next steps are segmentation and gene-aware correction of the predicted CNAs (Fig. 1). The only requirement for the selection of diploid control samples is that they should be processed using the same target selection kit as the tumor samples.

### 2.1 Datasets

To validate our method, we used three experimental amplicon sequencing datasets: A, B and C. The high amplicon density tumor DNA for datasets A and B was subjected to amplicon sequencing with target selection using the Ion Xpress™ Plus Fragment Library Kit by Life Technologies (panel of 406 genes including >6500 exons covered by >15 000 amplicons). Dataset C is a part of the SHIVA clinical trial (Le Tourneau *et al.*, 2012). This dataset was generated using the Ion AmpliSeq™ Cancer Panel V1 in combination with the Ion AmpliSeq™ Library Kit 2.0 by Life Technologies; for our analysis, we used 11 genes covered by 96 amplicons. We used 15 diploid samples (high amplicon density, control dataset X) and 6 diploid samples (low amplicon density, control dataset Y) to create the baseline for the normalization of datasets A/B and C, respectively.

In addition to amplicon sequencing, the tumor samples from dataset A were analyzed using an array CGH technique. Tumor samples from dataset C were processed with SNP arrays. The details of the processing of array CGH and SNP array data are provided in the Supplementary Materials.

### 2.2 Read counting

In the analysis of high-throughput sequencing data, an appropriate definition of read counts (RCs) is of high importance. In whole-genome sequencing data, RC refers to the number of reads starting in a given window (Boeva *et al.*, 2011). In WES and RNA sequencing data analysis, RC corresponds to the number of read mappings overlapping each exon (Amarasinghe *et al.*, 2013). When dealing with amplicon sequencing data, one could apply a similar approach and reasoning in terms of exons. However, this may lead to a loss of information. In amplicon sequencing, one exon can be targeted using two or more closely located or overlapping amplicons; averaging this information will decrease the sensitivity of CNA detection. In ONCOCNV, we therefore implemented an intuitive procedure to calculate RCs: each read is assigned to only one amplicon region, the one with which the read alignment has the maximum overlap. In the case of highly overlapping amplicons, it can be unclear which amplicon generated the read. We therefore merge amplicons when two of them overlap each other by >75% of their lengths.

Given that the amplicon sequencing reads are single ended and sequencing depth is extremely high, we do not discard duplicate reads to avoid distorting the RC disproportionately (Zhou *et al.*, 2014).

### 2.3 Normalization of RCs per target

The RC processing method implemented in ONCOCNV includes normalization for library size, GC content of each amplicon region and amplicon length. We call these kinds of biases ‘library specific’. The exact shape of the functional dependency of the RC on the GC content and amplicon length may be different in each library (Supplementary Figs S2A, B and S3A, B).

#### 2.3.1 Normalization with respect to library size

The average number of reads from a library mapped to an amplicon is Avg = N/R, where N is the total number of reads and R is the total number of amplicons. We normalize the raw read count (RRC) by the library size: $NRC_{Lib}=RRC/Avg$, where $NRC_{Lib}$ stands for the RC normalized with library size. Under the assumption of comparable efficiency of PCR amplification for all regions targeted, the RRC values would be similar for different amplicons, and thus, the normalized read count (NRC) values would typically be close to 1 (Supplementary Fig. S4).

#### 2.3.2 Normalization with respect to CG-content

**Fig. 2.**
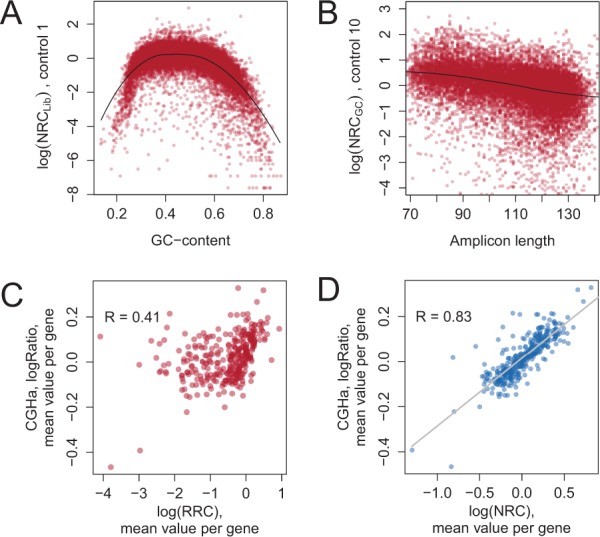
Normalization of RC data improves correlation with array CGH measurements. (**A**) GC-content bias. The *y*-axis shows the RC normalized with respect to library size (Sample X1). (**B**) Amplicon length bias. The *y*-axis shows the RC normalized with respect to GC-content and library size (Sample X10). (**C**) Correlation between RRCs and log-ratio values of array CGH (Sample A1). (**D**) Correlation between NRCs and log-ratio values of array CGH (Sample A1). In (C) and (D), mean value per gene is shown

For target sequencing data, as for whole-genome sequencing data (Boeva *et al.*, 2011), we observe a significant GC-content bias (Fig. 2A and Supplementary Fig. S2A). The shape of the GC-content dependency may vary even between datasets generated within the same laboratory (Supplementary Fig. S2B). We correct for the GC-content bias by using local polynomial regression fitting [LOESS (Cleveland *et al.*, 1992), R package stats, degree = 2]. The resulting values $NRC_{GC}$ do not depend on the GC content of the amplicon sequences (Supplementary Fig. S2C).

#### 2.3.3 Normalization with respect to target length

We observed that smaller amplicons usually generate more RCs than larger ones, probably owing to more efficient PCR amplification for smaller DNA fragments (Supplementary Fig. S2B and S3A). Similar to the GC content, this bias can be more or less pronounced in different samples (Supplementary Fig. S3B). We correct for the amplicon length bias by using the same technique as for the GC-content correction, i.e. LOESS (Cleveland *et al.*, 1992). The resulting values $NRC_{Len}$ do not depend on the GC content (Supplementary Fig. S3C) or on the length (Supplementary Fig. S3D) of the amplicon sequence.

### 2.4 Establishment of baseline

In addition to the library-specific bias, there is a technology-specific bias. This bias, even after normalization for the library-specific bias, is present to a different extent in the RCs (Supplementary Fig. S5A and B). Therefore, for each technological platform, we construct a baseline that reflects the technological bias in the diploid control samples and use it for the normalization of test samples.

To construct the baseline, we apply principal component analysis (PCA) to the NRCs $NRC_{Len}$ from the diploid samples. The first principal component (PC1) captures the most variation in *NRC_Len_* and highly correlates with the average value of *NRC_Len_* over the control samples (Pearson correlation 0.99, Supplementary Fig. S6). We observed that it was important to keep the second and third PCs, as they capture additional biases (Supplementary Fig. S5C). In our example (datasets A and B), the first three components account for 90.5% of the total variance in the NRCs of the diploid control samples, whereas the first component explains only 79% of the variance (Supplementary Fig. S6A). Thus, by default, we keep the first three PCs as baseline. This parameter may, however, be changed by the user after examination of the explained variance in the output of ONCOCNV. Also, if the number of control samples (*n*) is less than four, only (*n −* 1) PCs will be kept. At least two control samples are necessary to run ONCOCNV. When several tumor/matched normal datasets are present, the baseline should be created using all the control samples available.

For the final normalization of the sample RCs, we use a linear regression over the baseline. The resulting profile, *NRC_Final_*, shows lower variance than *NRC_Len_* and no directional bias (Supplementary Fig. S5D).

It should be noted that independent component analysis (ICA) could seem more appropriate than PCA to deal with the technology-specific bias. Each independent component could represent an independent source of noise in the experiment. The use of ICA instead of PCA will not, however, lead to different results in normalization based on the regression over the first components, as the first independent components are linear combinations of the first PCs (Hyvärinen and Oja, 2000).

### 2.5 Correction of control sample RCs for gender

A control dataset is often generated from a mixture of male and female samples. To correct the RCs in the control samples for gender, we apply the following strategy. After the first normalization step (normalization with respect to library size), we calculate $M_{i}=mean(NRC_{Len}\;on\;chrX)/mean(NRC_{Lib})$ for each control sample *i*. Then we choose the optimal model with one or two components for *M_i_*, according to the Bayesian information criterion for expectation–maximization initialized by hierarchical clustering for parameterized Gaussian mixture models (Fraley and Raftery, 2002). If the best model includes two components, we can conclude that our dataset represents a mixture of male and female samples: samples falling in a cluster with smaller *M_i_* are male and samples falling in a cluster with higher *M_i_* are female. If the best model contains only one cluster, we can conclude that all samples have the same gender and we assign male gender to them if the *M* value for all samples is <0.9. For all control samples annotated as male, we multiply the RCs on chrX by two to make them comparable with female control samples.

### 2.6 Assessment of standard deviation for NRCs

Although we know how to normalize sample RCs, we do not know which deviation from zero level should be called significant to call a copy number change. To solve this issue, we need to evaluate the standard deviation of *NRC_final_* for each amplicon region. We notice that different amplicon regions tend to have different standard deviations (Supplementary Fig. S7A). Generally, the higher the NRC (i.e. PC1), the lower the variance (Supplementary Fig. S7B). Further, we assume that *NRC_final_* follows a normal distribution, with the mean value depending on the copy number status (μ=0 in the case of copy number neutral), and the standard deviation depending on the first PC of our baseline (σ=f(PC1)) (Li *et al.*, 2012). To model σ=f(PC1), we use all control samples. However, the overall variance can be higher in some samples than in others (Supplementary Fig. S7). Thus, before the evaluation of σ=f(PC1), we rescale the control samples so that they have the same overall variance of 1. We model function f(PC1) using LOESS (degree = 2) (Supplementary Fig. S8) (Cleveland *et al.*, 1992). At the end of this step, each amplicon *i* is assigned standard deviation $\sigma_{i}$, which shows how the NRC for this amplicon varies in comparison with NRCs of other amplicon regions.

### 2.7 Processing of tumor sample RCs

Logarithmic values of the tumor sample RCs are normalized by library size, GC content and amplicon length. For further analysis, we keep residuals of the linear regression of the tumor NRCs over the baseline calculated for the control samples.

The resulting profiles are segmented using the circular binary segmentation (CBS) method [R packages PSCBS and DNAcopy (Olshen *et al.*, 2004; Venkatraman and Olshen, 2007)]. To avoid breakpoints at outlier values, for each amplicon *i*, we give the segmentation algorithm weightings *w*, which are inversely proportional to the variances $\sigma_{i}^{2}$. Generally, all NRC values within a segment resulting from the CBS correspond to a particular copy number status.

To annotate a segment as a gain, loss or neutral copy number, we define a segmentation and clustering approach. This is based on the idea that the mean values of NRCs of segments (segment mean values) should roughly correspond to integer copy number changes (Supplementary Fig. S9). In other words, the segment mean values corresponding to the same number of copies should cluster together. A similar approach was used by Gusnanto *et al.* (2012) to detect CNAs in whole-genome sequencing data.

Using R package mclust (Fraley *et al.*, 2012), we cluster weighted means of segment log ratio values. To prevent clustering errors, we add Gaussian noise to the data (Supplementary Fig. S9B and C). For each segment, the standard deviation of the added random variable is equal to the standard error of the mean, and thus depends on the length of the segment. Our approach is nearly identical to the classic non-parametric density estimation using the Parzen window method (Parzen, 1962). Here, to be able to apply the existing clustering tool mclust, we perform sampling from normal distribution instead of kernel density smoothing.

We select the level with the maximum density to be the neutral copy number level (zero level in Supplementary Fig. S9C). We center the ratio values on the zero level. All segments with mean values clustered with zero are further considered copy neutral. We evaluate the overall standard deviation $\sigma_{}^{sample}$ of the sample using the copy-neutral regions. We rescale the standard deviation $\sigma_{i}^{sample}=\sigma^{sample}*\sigma_{i}$, where $\sigma_{i}^{sample}$ is the standard variation for amplicon *i* in a given tumor sample.

### 2.8 Statistical validation of candidate CNAs

For each segment annotated as a putative CNA at the previous step, we perform two tests to ensure that the observed deviation from the normal cannot be because of mere chance. We calculate the *P*-values of the fixed variance test and the *t*-test under the null hypothesis that $\log\left(NRC_{final}^{i}\right)$ are generated by a normal distribution with mean value zero and standard deviation $\sigma_{i}^{sample}$.

#### 2.8.1 Fixed variance test

If, for a copy-neutral amplicon *i* values,

$$X_{i}=\log\left(NRC_{final}^{i}\right)/\sigma_{i}^{sample}$$

follows a normal distribution N(0,1), then the arithmetic mean of a sample $X_{1},X_{2},X_{3},\ldots ∼N(0,1)$ is also normally distributed: X¯∼N(0,1n). To test the significance of a deviation from neutrality for a consecutive set of target regions k+1…,k+n, we use the following statistics formula:

$$abs\left(\left(\sum_{i=k+1}^{i=k+n}\log\left(NRC_{final}^{i}\right)/\sigma_{i}^{sample}\right)/n\right),$$

which should follow half-normal distribution with $\theta =\sqrt{\pi n}/\sqrt{2}$.

#### 2.8.2 T-test

Under the hypothesis that target regions i=k+1…,k+n are copy-neutral random variables, $X_{i}$ are normally distributed with expectation 0. Thus, we can apply a *t*-test to test the hypothesis about the mean value of ${\left\{X_{i}\right\}}_{i=k+1\ldots ,k+n}$. It should be noted that, as the standard deviation is not fixed but evaluated via the sample standard deviation, the *t*-test usually produces higher *P*-values than the fixed variance test.

#### 2.8.3 Filtering of candidate CNAs

We keep only those candidate CNAs for which the fixed variance test and *t*-test *P*-values are <0.01. This allows us to be extremely stringent in filtering false-positive CNAs (Supplementary Fig. S10).

Contamination by normal cells, tumor hyper-ploidy and presence of subclones deteriorates the copy number ratios and impede the accuracy of CNA detection. Unfortunately, it is impossible to get reliable information about these factors from amplicon sequencing data. Thus, we imposed a detection threshold on the number of cells with a given CNA. The expected NRC value in a region of a gain or loss of *l* copies can be calculated as ExpectedNRC=(1−c)·1+c·(1±l/P), where *c* is the fraction of all cells in the sample containing the CNA and *P* is the tumor ploidy. The higher the tumor ploidy and contamination by normal cells and the lower the fraction of tumor cells containing each CNA, the closer the expected NRC will be to 1, and the more difficult it will be to discriminate between true and false calls (Supplementary Fig. S11A). To avoid false-positive predictions, ONCOCNV filters out all candidate CNAs whose weighted geometric mean of NRCs varies within the range (0.875 to 1.125). This filter is likely to remove all CNAs present in fewer than 25, 33 and 50% of cells in diploid, triploid and tetraploid tumors, respectively (Supplementary Fig. S11B).

### 2.9 Detection of one-point copy number changes

Our method does not focus on the identification of one-point copy number changes (gain or loss of one exon/amplicon region). Nevertheless, we provide a functionality to output *P*-values and outlier statuses for each target region *i* based on a normal distribution $N\left(wm_{seg},\sigma_{i}^{ratio}\right)$ of ratio values, where $wm_{seg}$ is the weighted mean of the large segment including *i*, and $\sigma_{i}^{ratio}$ is the evaluated standard deviation. Similar to CONTRA (Li *et al.*, 2012), we adjust these *P*-values for multiple testing using the Benjamini and Hochberg correction (Benjamini and Hochberg, 1995).

### 2.10 Readjustment of segmentation with a gene-aware model

The segmentation procedure described above is performed in a gene-unaware way. Although segmentation usually places a breakpoint between two genes, in some cases, the breakpoint is positioned within a gene (Supplementary Fig. S12). In this case, there are two possible scenarios: (i) the chromosomal break occurs within the gene or (ii) because of noise, the predicted breakpoint was mis-positioned several probes left or right of the real breakpoint. We developed a readjustment strategy to account for the second scenario. When a breakpoint falls within the gene and breaks it into several (usually two) segments, we test whether the mean value of one of the segments could explain all the observed log(NRC) values of the gene (*t*-test). A gene with more amplicon regions is more likely to be correctly annotated with respect to the copy number status than a gene with fewer amplicon regions.

## 3 RESULTS

### 3.1 Comparison with WES-specific methods: NextGENe and ADTEx

We decided to compare our CNA calling method in ultra-deep targeted sequencing data with two WES-specific tools: ADTEx (Amarasinghe *et al.*, 2013), a method based on hidden Markov models (HMMs) for CNA calling in targeted (exome) sequencing data, and NextGENe, commercial software designed by SoftGenetics. Although both ADTEx and NextGENe were tested on WES data only, conceptually there is no reason why these methods should not be applied to a smaller panel of targeted genes (∼400 in our case). We applied ONCOCNV, ADTEx and NextGENe to a high amplicon depth sequencing dataset of eight tumor samples. The array CGHs performed for these samples showed different CNA complexity levels (Supplementary Fig. S13). Although we also compared our method with CONTRA (Li *et al.*, 2012)—a method to detect one-exon copy number changes as well as large CNAs in targeted sequencing data—we do not report the results of this comparison in this article as, in our data, CONTRA identified <10% of the true CNAs (Supplementary Fig. S14).

Until now, ADTEx has not allowed the creation of a baseline using several control files. We therefore applied two possible strategies: (i) we selected 1 of 15 control samples and used it as a matched normal control and (ii) we merged the 13 control samples in 1.AM file and used it a matched control. In the ADTEx manual, it is advised to discard duplicate reads before running the analysis. In our case, duplicate reads constitute ∼95% of all reads (because of the high depth of coverage of our data). We therefore applied two independent strategies to call CNAs with ADTEx: with and without duplicate filtering.

For the four output files (single and merged controls; with and without duplicates), we checked the correlation between the NRCs and log ratio values calculated during CGH experiments. The correlation values were similar for all four types of output file (Supplementary Table S1). Following recommendations from the authors of the ADTEx manual, we decided to use the ADTEx analysis based on one control sample including duplicate filtering (single control, no duplicates) for further comparison.

#### 3.1.1 Comparison of normalization results

**Fig. 3.**
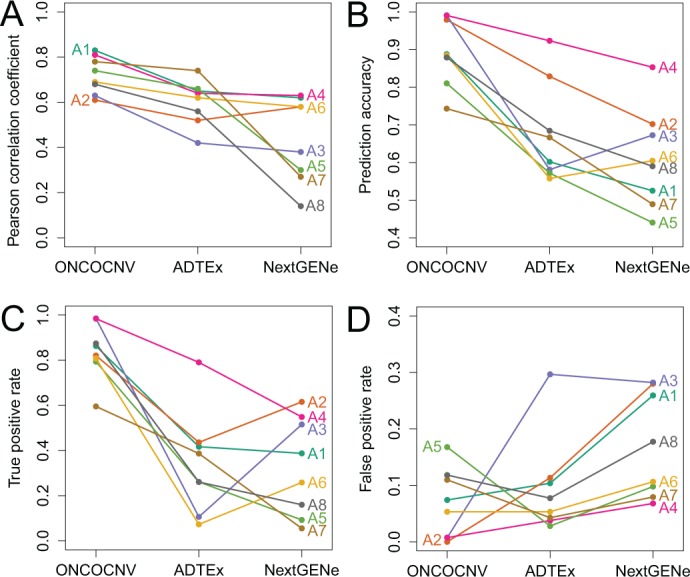
Comparison of RC normalization and accuracy of CNV calls achieved by ONCOCNV, ADTEx and NextGENe for samples from dataset A. (**A**) Correlation between NRCs and log ratio values of array CGH. (**B**) Prediction accuracy. Accuracy = (#True predictions)/(#All predictions), where each prediction corresponds to a gene copy number status (gain, neutral or loss). (**C**) True-positive rate. (**D**) False-positive rate

The values resulting from the ONCOCNV normalization procedure correlate highly with the log ratio values of the array CGH experiments performed on the same samples (Figs 2C, D and 3A, Supplementary Table S1). The correlation was higher for ONCOCNV NRCs than for RCs normalized by ADTEx and NextGENe. For example, for sample A1, the linear correlation between CGH log ratio values and ONCOCNV NRCs was 0.83, compared with 0.65 and 0.62 for ADTEx and NextGENe, respectively (Supplementary Fig. S15). This demonstrates that the normalization procedure used in ONCOCNV has clear advantages over those used in CNA calling methods developed for exome sequencing data analysis.

We confirmed that correction for the GC content plays a key role in amplicon data normalization (Supplementary Fig. S16). Correction for the technology-specific bias using the first PCs of PCA is also important to decrease the variance and correct for outliers in NRCs (Supplementary Figs S5 and S16C, D, G and H).

#### 3.1.2 Comparison of prediction accuracies

**Fig. 4.**
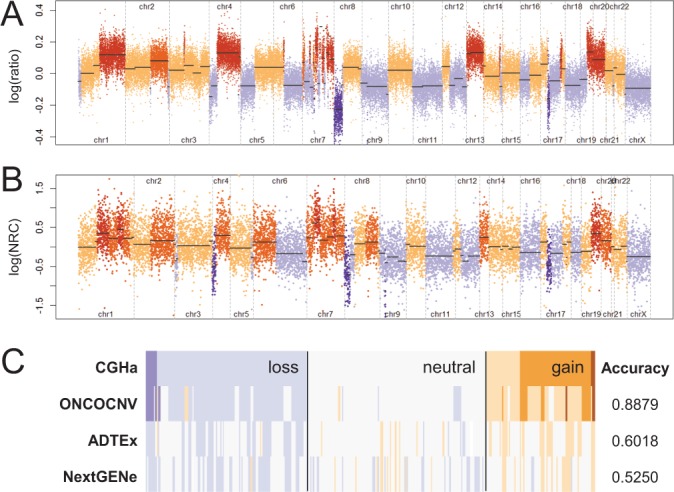
Example of CNVs called by ONCOCNV, ADTEx and NextGENe (Sample A1). (**A)** Array CGH profile for sample A1, segmented using cghseg: purple (loss), orange (gain). *x*-axis corresponds to probe indexes. (**B)** Copy number profile calculated by ONCOCNV. *x*-axis corresponds to amplicon indexes. (**C)** Agreement between CNVs predicted from array CGHs and amplicon sequencing. Each vertical bar denotes a gene copy number status: white (neutral), purple (loss) and orange (gain)

For all samples, ONCOCNV achieved much higher prediction accuracy than the other two tools (Figs 3B–D and 4 and Supplementary Table S2). Here, accuracy measures the proportion of genes with correctly annotated copy number status: neutral, gain or loss. The overall prediction accuracy varied from 0.74 to 0.99 for ONCOCNV, while it was significantly lower for the other two tools: 0.56–0.92 for ADTEx and 0.44–0.85 for NextGENe (Fig. 3B). For all methods, samples with a high number of genomic rearrangements were more challenging to analyze correctly than samples with less complex profiles (Supplementary Figs S17 and S18).

### 3.2 Validation on 30 ErbB2-positive tumor samples

To validate our method further, we applied ONCOCNV to analyze amplicon sequencing data generated for 30 ErbB2-positive ovarian cancer samples. The copy number status of the *ErbB2* gene was assessed via fluorescence *in situ* hybridization using FFPE tissue sections. The gene *ErbB2* (also known as *HER2*) encodes a receptor tyrosine kinase whose activation plays an important role in cell survival and drives cell proliferation (Tai *et al.*, 2010). *ErbB2* is frequently overexpressed in breast, gastric, ovarian and prostate cancer. *ErbB2* overexpression most often occurs because of a gene amplification (Pauletti *et al.*, 1996). Several drug compounds are used in clinics to block ErbB2 signaling in the case of overexpression of *ErbB2*. Thus, a personalized treatment is available for patients with amplification of the *ErbB2* gene (approved in clinical practice for breast and gastric cancer patients).

**Fig. 5.**
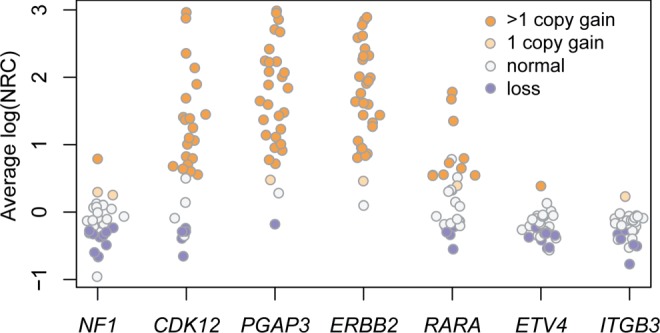
Accuracy of the detection of the *ErbB2* amplification. Normalized read count (NRC) and copy number prediction for the *ErbB2* gene and six adjacent genes in 30 Erbb2-positive ovarian cancer samples (dataset B). The Y-axis shows the average log(NRC) per gene. The color corresponds to the predicted copy number status: purple (loss), white (neutral), orange (gain). The darker orange color shows a gain of two and more copies of the gene

In 29 of the 30 ErbB2-positive samples, ONCOCNV predicted a gain in the genomic region containing the *ErbB2* gene (Fig. 5). Twenty-eight samples carry more than one additional copy of *ErbB2* (orange points in Fig. 5). The high true-positive rate (>96%) demonstrates the validity of our approach for the detection of amplification of actionable genes.

Intriguingly, in 1 sample of the 30, ONCOCNV did not detect any gain on chromosome 17 containing the *ErbB2* gene (Supplementary Fig. S19). The high quality of the corresponding NRC profile suggests to us that the duplication of ErbB2 may have happened in a subclonal population of cells that, for some reason, was not present in the tissue section subjected to amplicon sequencing. Unfortunately, no more of this sample was available and no further investigation was possible.

### 3.3 Validation on the SHIVA clinical trial dataset

We applied ONCOCNV to a low amplicon density dataset of tumors sequenced in the SHIVA clinical trial (Le Tourneau *et al.*, 2012). The data corresponded to a targeted sequencing of 190 amplicons covering the exons of 46 actionable genes. The number of amplicons varied from 1 to 17 per gene. As our algorithm was designed to deal with data containing multiple amplicon regions per gene, for our analysis, we kept 11 genes. Each of them contained at least seven amplicon regions. The 11 genes selected were located on 10 different chromosomes. In the SHIVA trial, a dual approach is used: amplicon sequencing is used to detect single nucleotide variants and indels, whereas SNP arrays provide information about the copy number status of the genes and contamination by normal cells. We wanted to test whether amplicon sequencing-based detection of CNAs could replace currently used SNP arrays.

As when using only 11 genes, it is often impossible to assess the correct zero level, we considered relative gains and losses in the calculation of CNA prediction accuracy (see Discussion). For example, if the SNP array technique detected a one copy loss of the first five genes whereas ONCOCNV predicted a one copy gain of the last six genes, we considered that the latter prediction was correct (the absolute prediction accuracy is shown in Supplementary Fig. S20).

**Fig. 6.**
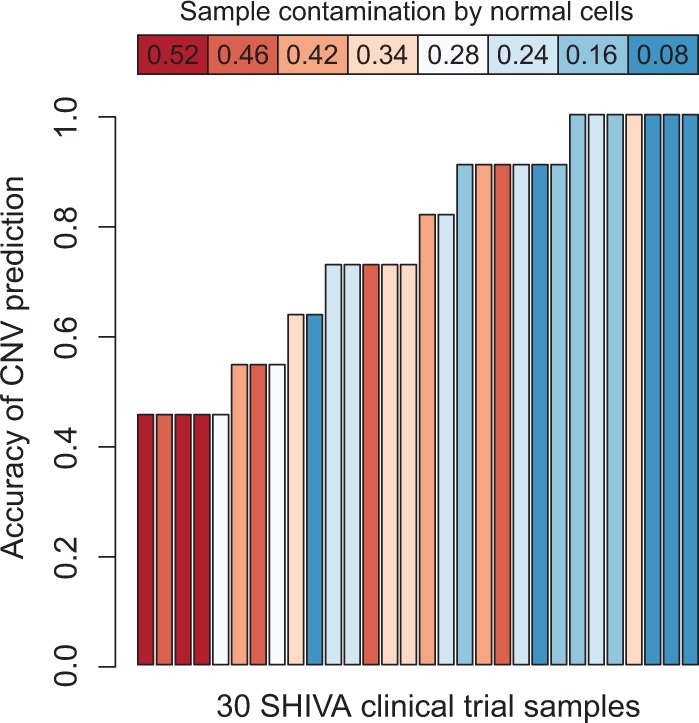
CNA detection accuracy in 30 samples from the SHIVA clinical trial (dataset C). The accuracy of CNA detection is negatively correlated with the percentage of normal cell contamination (Pearson *r* = −0.64, *P*-value = 10^−4^)

We observed a high accuracy of CNA detection for samples with low contamination by normal cells: for all but one sample with <18% contamination, the CNA detection accuracy was at least 90% (Fig. 6). As we expected, our ability to detect CNAs in low amplicon density datasets strongly depends on the normal contamination (Pearson correlation coefficient 0.64, *P*-value 10^−^^4^).

### 3.4 Data and software availability

Amplicon sequencing and CGH data and the ONCOCNV source code are available at http://oncocnv.curie.fr/

## 4 DISCUSSION

We proposed a method to analyze amplicon sequencing data to be used to detect CNAs involving at least several exons. Our method, ONCOCNV, demonstrated a high accuracy of CNA predictions for the samples for which we had the results of array CGH analysis as gold standard. ONCOCNV significantly outperformed methods developed for exome sequencing data analysis. This is because of the extensive normalization strategy, which includes normalization for the GC content, amplicon length (via LOESS correction) and other technology-specific biases (via PCA of the control samples).

An important advantage of ONCOCNV is the refinement of breakpoints falling initially within a gene (i.e. after the segmentation of NRC profiles). ONCOCNV reanalyzes the NRCs upstream and downstream of the putative breakpoint and decides whether intragenic breakpoint has indeed taken place and that it is not the result of inaccurate segmentation (Supplementary Fig. S12).

In our dataset, we observed samples (e.g. controls 3 and 9, Supplementary Fig. S2A) where a large fraction of amplicons (26% for both) have zero or extremely low RCs. We would like to point out that ONCOCNV is able to detect such artifacts and analyze CNAs correctly in the corresponding samples.

The prediction of gains and losses by ONCOCNV includes information on average log⁡(NRC). In addition, it estimates possible copy numbers. This estimate is based on the assumption that the tumor sample genome is diploid and that the sample is not contaminated by normal cells. We are aware that this assumption might be far from being true. However, we prefer to provide an estimate of the probable copy number to allow the user to distinguish between gains and amplifications, and between one and two allele losses. The question of predicting the exact copy number cannot be solved by using copy number profiles alone. The assessment of B allele frequencies provides a possible solution (Popova *et al.*, 2009). We did not use this solution in ONCOCNV because of the high dispersion of B allele frequencies in the amplicon sequencing data (data not shown).

CNAs detected by ONCOCNV (and by other software whose algorithm is based only on the analysis of copy number profile) are relative to the evaluated zero level. For instance, if we measure the copy numbers of only a pair of genes, we will not be able to see a gain or loss if this happens simultaneously in both genes. Thus, to properly estimate the zero level, many genes should be included in the targeted gene panel.

As we expected, we detected a fall in the CNA detection accuracy for samples that were highly contaminated by normal cells. In this situation, the noise in measurements is comparable with the expected difference between measurements in the case of one copy gain or loss. As our approach does not use information about B allele frequencies, certain combinations of normal contamination and tumor ploidy significantly impair the efficiency of the technique. This is especially true when there are a small number of measurements (amplicons) per gene.

We would like to point out that ONCOCNV does not detect one-amplicon amplifications/deletions. We believe the noise in current amplicon-based technologies does not allow detection of such CNVs with high certainty. However, there are methods, such as CONTRA (Li *et al.*, 2012), which predict the CNVs of single amplicon regions.

We do not position ONCOCNV as a tool for exome sequencing data analysis. Unlike many exome sequencing data analysis tools, ONCOCNV does not take into account B allele frequencies. Because of this limitation, ONCOCNV is not able to evaluate the level of the contamination by normal cells or improve the accuracy of CNA calling by simultaneously processing copy number and B allele frequency profiles (Boeva *et al.*, 2012; Sathirapongsasuti *et al.*, 2011).

Our analysis of biases intrinsic to amplicon sequencing revealed two factors influencing the RCs: amplicon length and GC content. As LOESS correction is possible only for the amplicon sizes frequently present in the dataset (from 70 to 140 bp in our case), we advise using as narrow a range of amplicon lengths as possible in the future design of amplicon regions. Similarly, for the GC-content normalization, the CNA prediction may be inaccurate for amplicons with extremely high or low GC content. This is especially true in the case when all amplicon regions within a gene (usually overlapping with exons) have an extremely high or low GC content. For such genes, we advise designing additional amplicons in the intronic regions with moderate GC content if it is planned to completely bypass array CGH or a similar technique to predict the copy number status of the targeted regions.

## 5 CONCLUSIONS

We present ONCOCNV, a computational method and software tool to detect CNAs in tumor amplicon sequencing data. Our method includes normalization of RCs for library-specific biases (library size, GC content and amplicon length) and for technology-specific bias. To normalize for the latter, we use a set of control samples. We demonstrate that ONCOCNV significantly outperforms methods such as ADTEx (Amarasinghe *et al.*, 2013) and NextGENe developed for the analysis of exome sequencing data. To make ONCOCNV useful for both bioinformaticians and clinicians, we output both detailed and summarized results, as well as a visualization of the annotated copy number profile (Fig. 4B). We believe that the use of our method on large panels of targeted genes could potentially replace CGH or SNP arrays in the future.

*Funding*: The SHIVA trial is supported by the grant ANR-10-EQPX-03 from the Agence Nationale de le Recherche (Investissements d’avenir) and SiRIC (Site de Recherche Intégré sur le Cancer). High-throughput sequencing was performed by the NGS platform of the Institut Curie, supported by grants ANR-10-EQPX-03 and ANR10-INBS-09-08 from the Agence Nationale de la Recherche (Investissements d’avenir) and by the Canceropôle Ile-de-France.

*Conflict of Interest*: none declared.

## Supplementary Material

Supplementary Data
