# Supplementary material for: Multi-factor data normalization enables the detection of copy number aberrations in amplicon sequencing data
Source: Bioinformatics. 2014 Jul 12;30(24):3443–50. doi: 10.1093/bioinformatics/btu436 (PMC4253825; doi:10.1093/bioinformatics/btu436)
Supplement: Supplementary Data [file supp_30_24_3443__index.html]

Multi-factor data normalization enables the detection of copy number aberrations in amplicon sequencing data — Multi-factor data normalization enables the detection of copy number aberrations in amplicon sequencing data — Multi-factor data normalization enables the detection of copy number aberrations in amplicon sequencing data — Supplementary Data 

# Multi-factor data normalization enables the detection of copy number aberrations in amplicon sequencing data

## Supplementary Data

files

**Files in this Data Supplement:**

- Supplementary Data - pdf file
