# Supplementary material for: Multi-factor data normalization enables the detection of copy number aberrations in amplicon sequencing data
Source: Bioinformatics. 2014 Jul 12;30(24):3443–50. doi: 10.1093/bioinformatics/btu436 (PMC4253825; doi:10.1093/bioinformatics/btu436)
Supplement: Supplementary Data [file supp_btu436_boeva_Supplementary_Materials.pdf]

Supplementary Materials

Multi-factor data normalization enables the detection of copy number aberrations in amplicon sequencing data

Boeva *at al*.

Supplementary methods:

Processing of validation array CGH and SNP array data

Supplementary Figures 1-20:

Supplementary Figure 1. Summary of amplicon sequencing technique

Supplementary Figure 2. GC-content bias in the amplicon sequencing data

Supplementary Figure 3. Amplicon length bias in the amplicon sequencing data

Supplementary Figure 4. Distribution of read counts normalized with regard to library size (NRC_{Lib})

Supplementary Figure 5. Baseline creation

Supplementary Figure 6. Properties of principal components extracted from normalized read counts for control samples

Supplementary Figure 7. Variance in NRC_{final} values depends on first principal component (PC1) values

Supplementary Figure 8. LOESS fitting for $\sigma = f(PC1)$

Supplementary Figure 9. Segmentation and clustering approach

Supplementary Figure 10. ONCOCNV uses two statistical tests to prevent the prediction of false positive copy number alterations

Supplementary Figure 11. Theoretical sensitivity of CNA detection by ONCOCNV

Supplementary Figure 12. Segmentation in a gene-unaware way can misplace breakpoints

Supplementary Figure 13. Array CGH copy number profile for samples A1-A8, segmented using cghseg

Supplementary Figure 14. Agreement between CNVs predicted from CGH arrays and amplicon sequencing by CONTRA software

Supplementary Figure 15. Correlation between log ratio values of array CGH and read counts normalized by ONCOCNV, ADTEx and NextGENe

Supplementary Figure 16. Relative importance of different normalization steps in ONCOCNV

Supplementary Figure 17. Comparison of CNVs called by ONCOCNV, ADTEx and NextGENe with predictions based on array CGH data for a low complexity profile (Sample A4)

Supplementary Figure 18. Comparison of CNVs called by ONCOCNV, ADTEx and NextGENe with predictions based on array CGH data for a high complexity profile (Sample A5)

Supplementary Figure 19. Visualization of normalized read count profile for ErbB2-positive sample showing no ERBB2-gain when analyzed by ONCOCNV

Supplementary Figure 20. Absolute CNV detection accuracy in 30 samples from the SHIVA clinical trial

Supplementary Tables:

Supplementary Table 1. Correlation between log ratio values of arrays CGH and read counts normalized by different methods

Supplementary Table 2. CNV prediction accuracy

Processing of validation array CGH and SNP array data

Array CGH processing

DNA extraction, sample preparation and hybridization were carried out according to the manufacturer's protocol for formalin-fixed paraffin-embedded (FFPE) samples (Protocol G4410-90020, Version 3.4, July 2012, Agilent Technologies). Human genomic DNA from Promega (Male - #G147A, Female - #G152A) was used as reference genome. Array CGH analyses were performed using Sureprint G3 unrestricted CGH 8X60K microarrays (#G4827A, Agilent Technologies), which were scanned with an Agilent Type C scanner (G2505C - DNA microarray scanner with SureScan high-resolution technology) using Agilent Scan Control software Version A.8.4.1. Feature Extraction software (Version 11.0.1.1, Agilent Technologies) with an hg19 annotated design file (025683_D_F_20100809, Agilent Technologies) and a modified extraction protocol (CGH_1100_Jul11, provided by Agilent Technologies) were used to extract raw data from tiff files obtained after scanning. The array-based CGH data were analyzed with Agilent Genomic Workbench 7.0.4.0 (Agilent Technologies). The log ratio values were segmented using R package CGHseg (Picard *et al.*, 2011). The SCA algorithm (unpublished) was used to detect significantly aberrant genomic regions.

SNP array processing

SNP array experiments were performed using a CytoScan® HD Array Kit according the manufacturer's protocol (Affymetrix). 250 ng of genomic DNA were used for the target preparation and hybridized microarrays. When the quantity of genomic DNA available was less than 250 ng, a first whole genome amplification (Qiagen, REPLI-g Mini Kit PN: 150023) step was carried out before the assay. The CytoScan® HD profiles were normalized with the Affymetrix Power Tools software package (http://www.affymetrix.com). The log R ratio profile was then segmented in order to detect breakpoints and assign copy number status using Colibri (Rigaill, 2010) and GLAD (Hupé *et al.*, 2004) software. The detection of CNAs was performed with the GAP algorithm (Popova *et al.*, 2009). The sample cellularity and tumor ploidy were estimated by GAP.

Picard,F. *et al.* (2011) Joint segmentation, calling, and normalization of multiple CGH profiles. *Biostatistics*, **12**, 413–428.

Hupé,P. *et al.* (2004) Analysis of array CGH data: from signal ratio to gain and loss of DNA regions. *Bioinformatics*, **20**, 3413–3422.

Rigaill,G. (2010) Pruned dynamic programming for optimal multiple change-point detection. *ArXiv10040887 Stat*.

Supplementary Figures

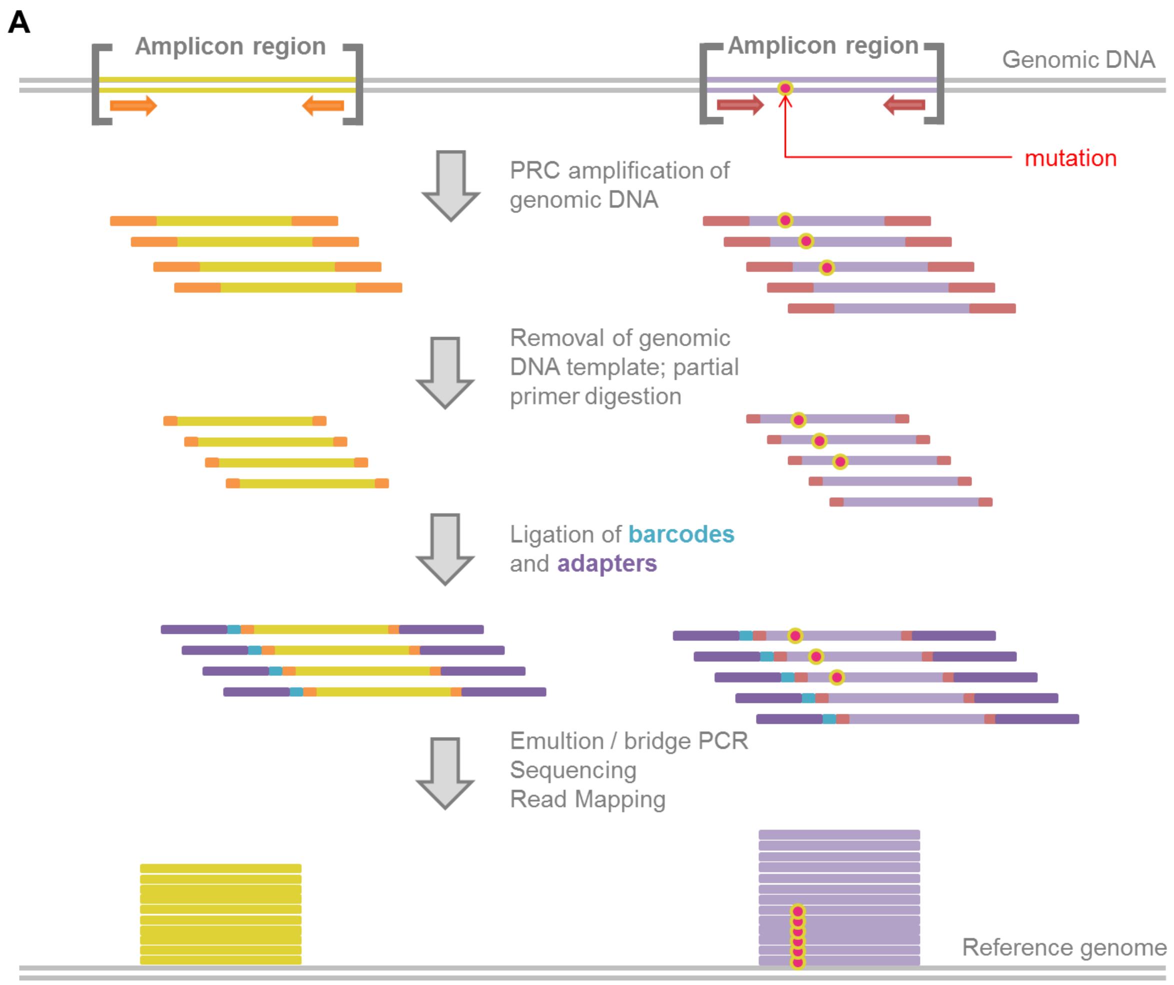

B

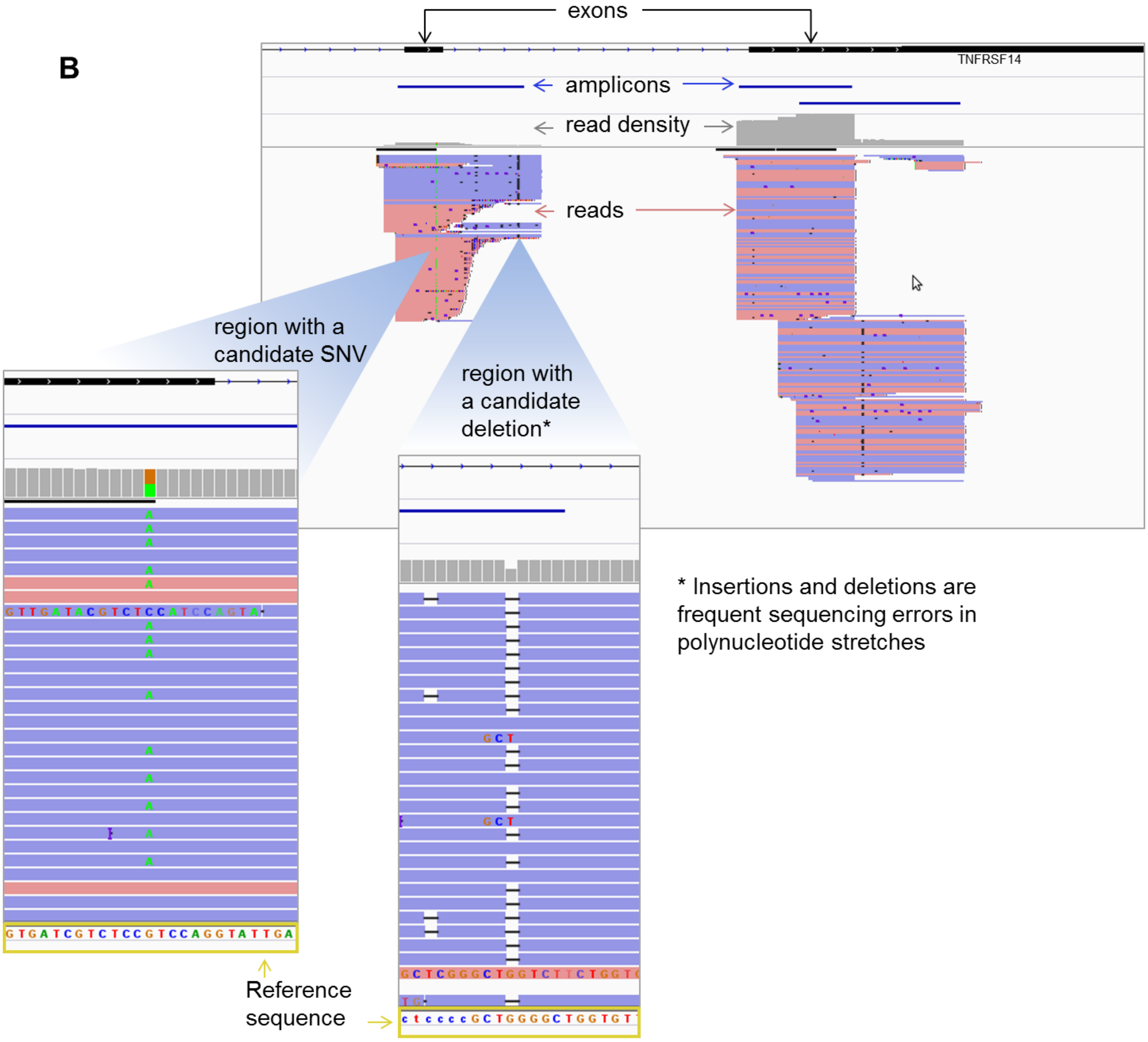

Supplementary figure 1. Summary of amplicon sequencing technique

A. Main steps of amplicon sequencing technique. Currently, in amplicon sequencing DNA fragments are sequenced with the low input/low output sequencing technology (e.g., Ion Proton by Life Technologies). When this article is written, the sequencers of this type are the most appropriate for sequencing small amount of reads (~1-2 millions) at low cost and high speed. Reads sequenced with these technologies have various lengths. Since the total length of the targeted regions is relatively small (usually less than 2 Mb), the mapping of reads is fast and unambiguous. The user can use any mapping algorithm that allows mapping of reads with various lengths. **B**. Visualization of the typical configuration of read mappings (with Integrative Genomics Viewer). Exons 7 and 8 of the TNFRSF14 gene.

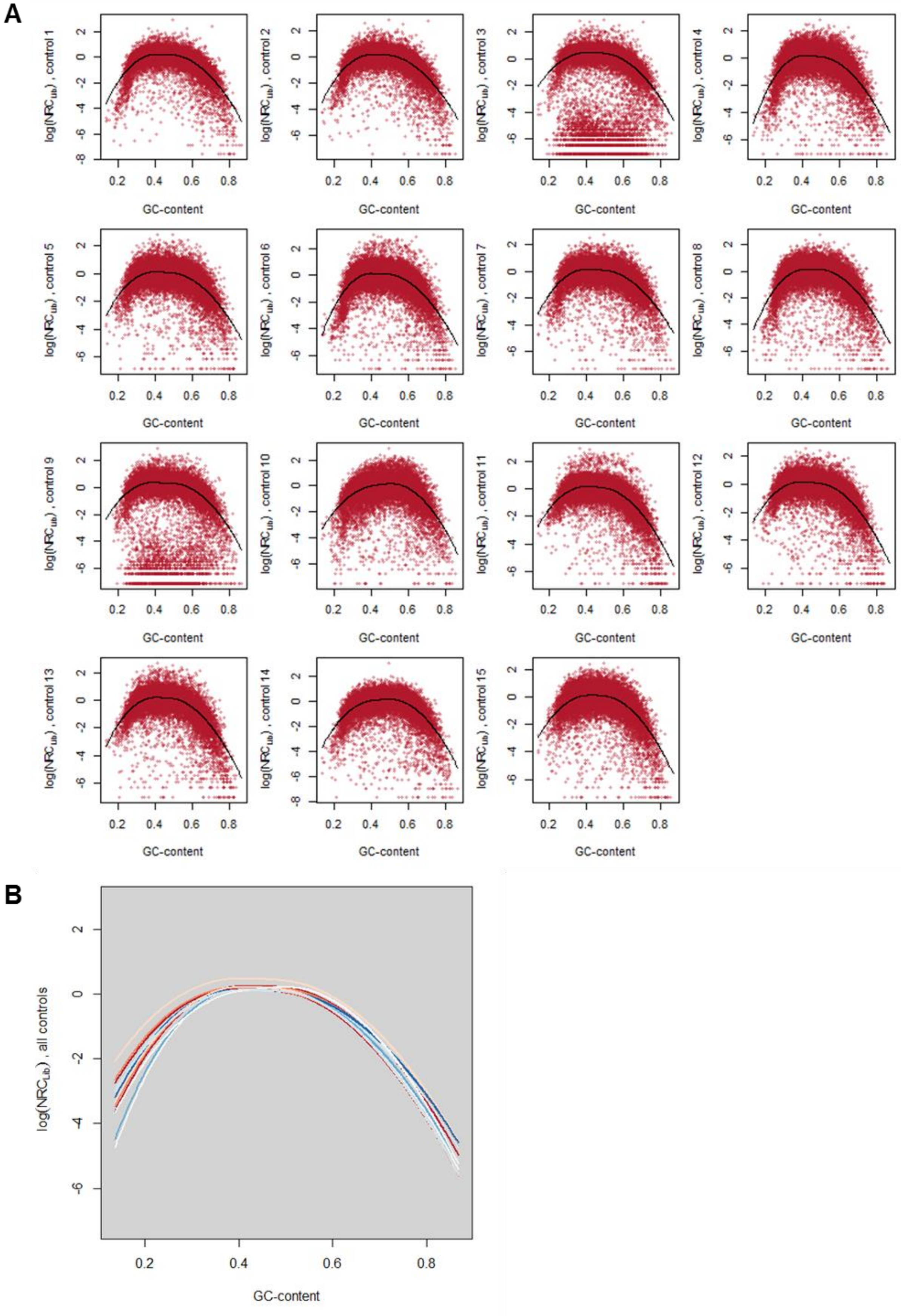

A
log(NRCLib), control 1
log(NRCLib), control 2
log(NRCLib), control 3
log(NRCLib), control 4
log(NRCLib), control 5
log(NRCLib), control 6
log(NRCLib), control 7
log(NRCLib), control 8
log(NRCLib), control 9
log(NRCLib), control 10
log(NRCLib), control 11
log(NRCLib), control 12
log(NRCLib), control 13
log(NRCLib), control 14
log(NRCLib), control 15
GC-content
B
log(NRCLib), all controls
GC-content

C

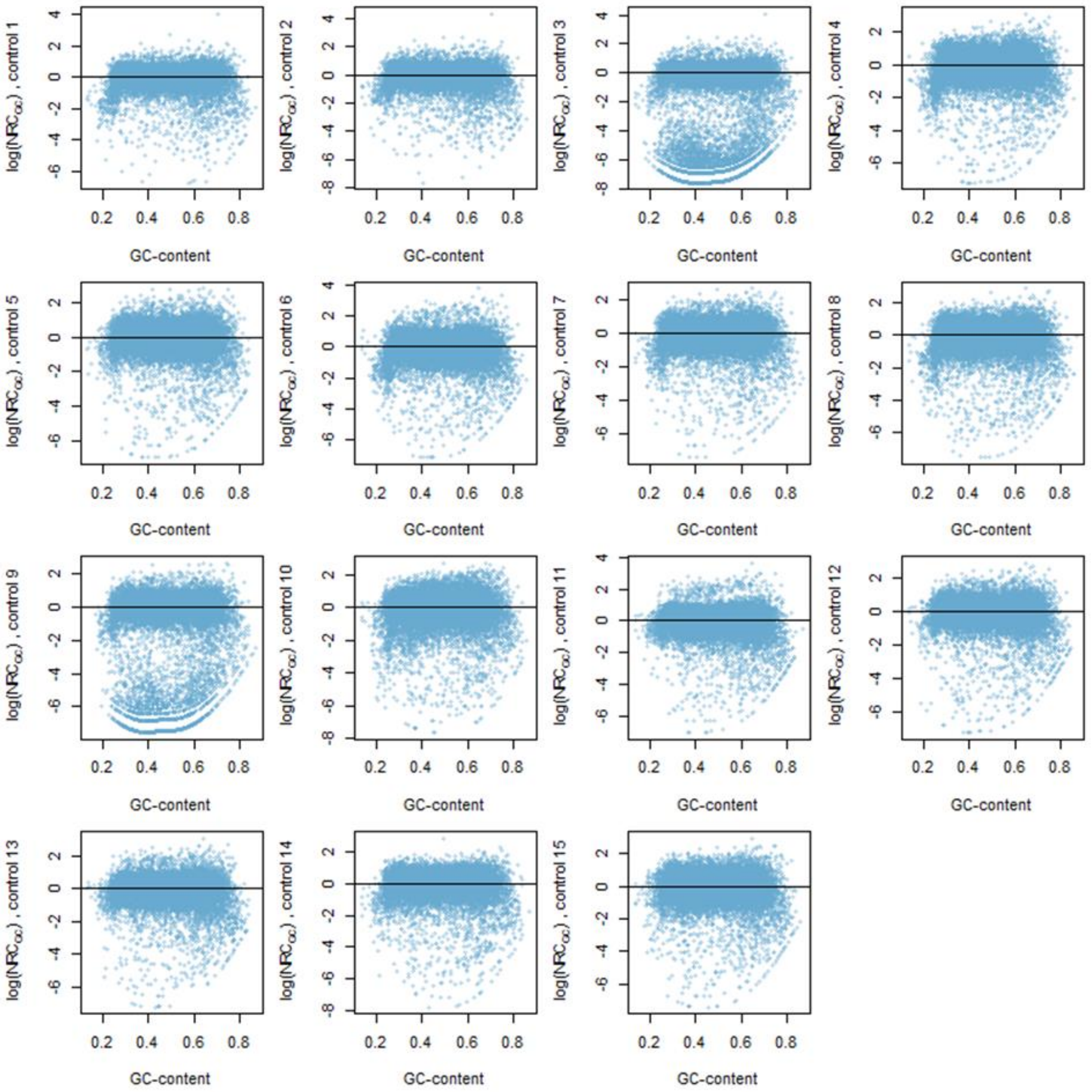

Supplementary Figure 2. GC-content bias in the amplicon sequencing data (dataset X)

A. Logarithms of read count normalized with regard to library size (log(NRC_{Lib})) *versus* GC-content. **B**. Fitted values of GC-content functional dependency for 15 control samples. **C**. Logarithms of read count normalized with regard to GC-content (log(NRC_{GC})).

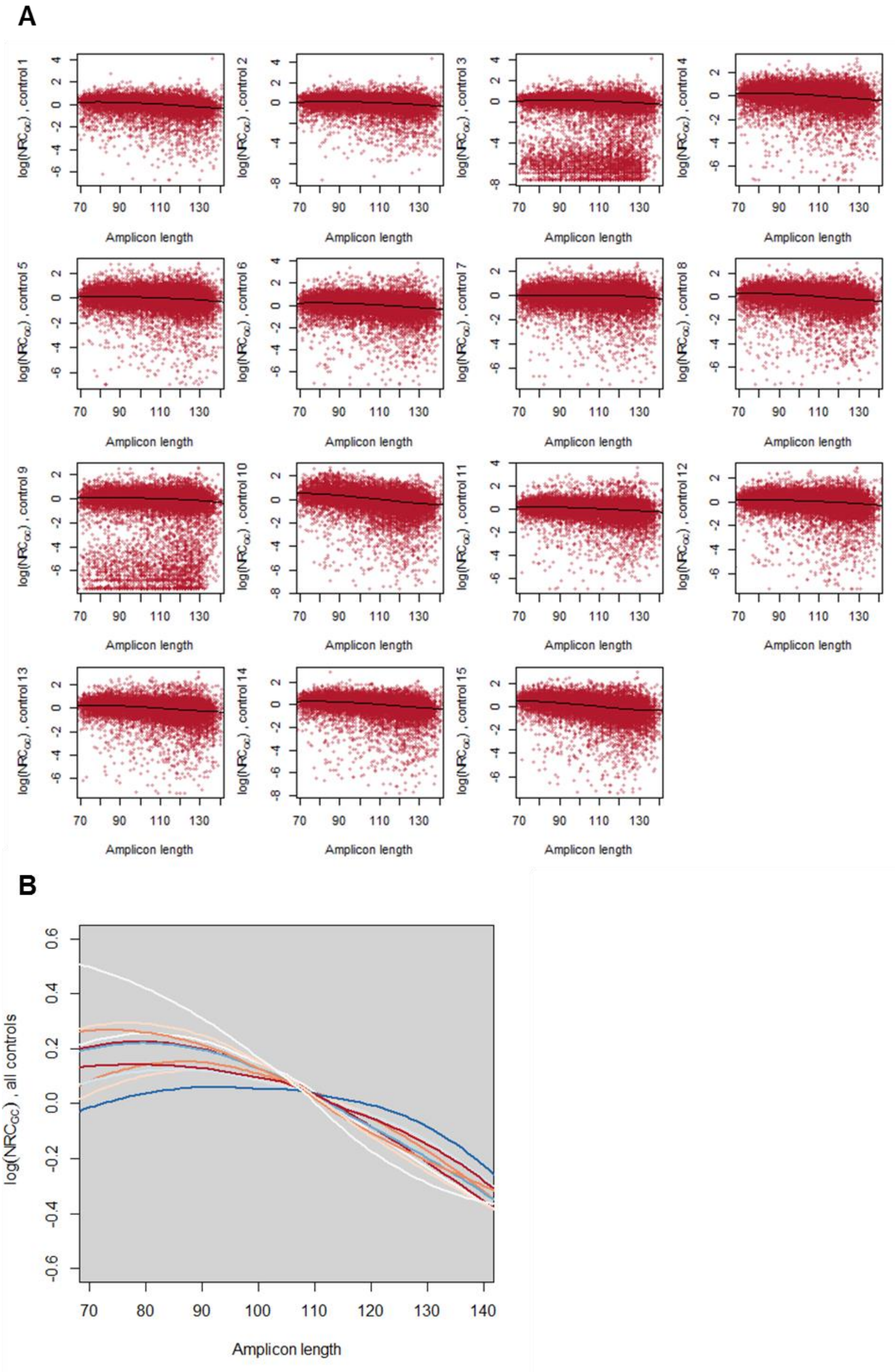
A
log(NRC_GC) , control 1
log(NRC_GC) , control 2
log(NRC_GC) , control 3
log(NRC_GC) , control 4
log(NRC_GC) , control 5
log(NRC_GC) , control 6
log(NRC_GC) , control 7
log(NRC_GC) , control 8
log(NRC_GC) , control 9
log(NRC_GC) , control 10
log(NRC_GC) , control 11
log(NRC_GC) , control 12
log(NRC_GC) , control 13
log(NRC_GC) , control 14
log(NRC_GC) , control 15
Amplicon length
B
log(NRC_GC) , all controls
Amplicon length

C

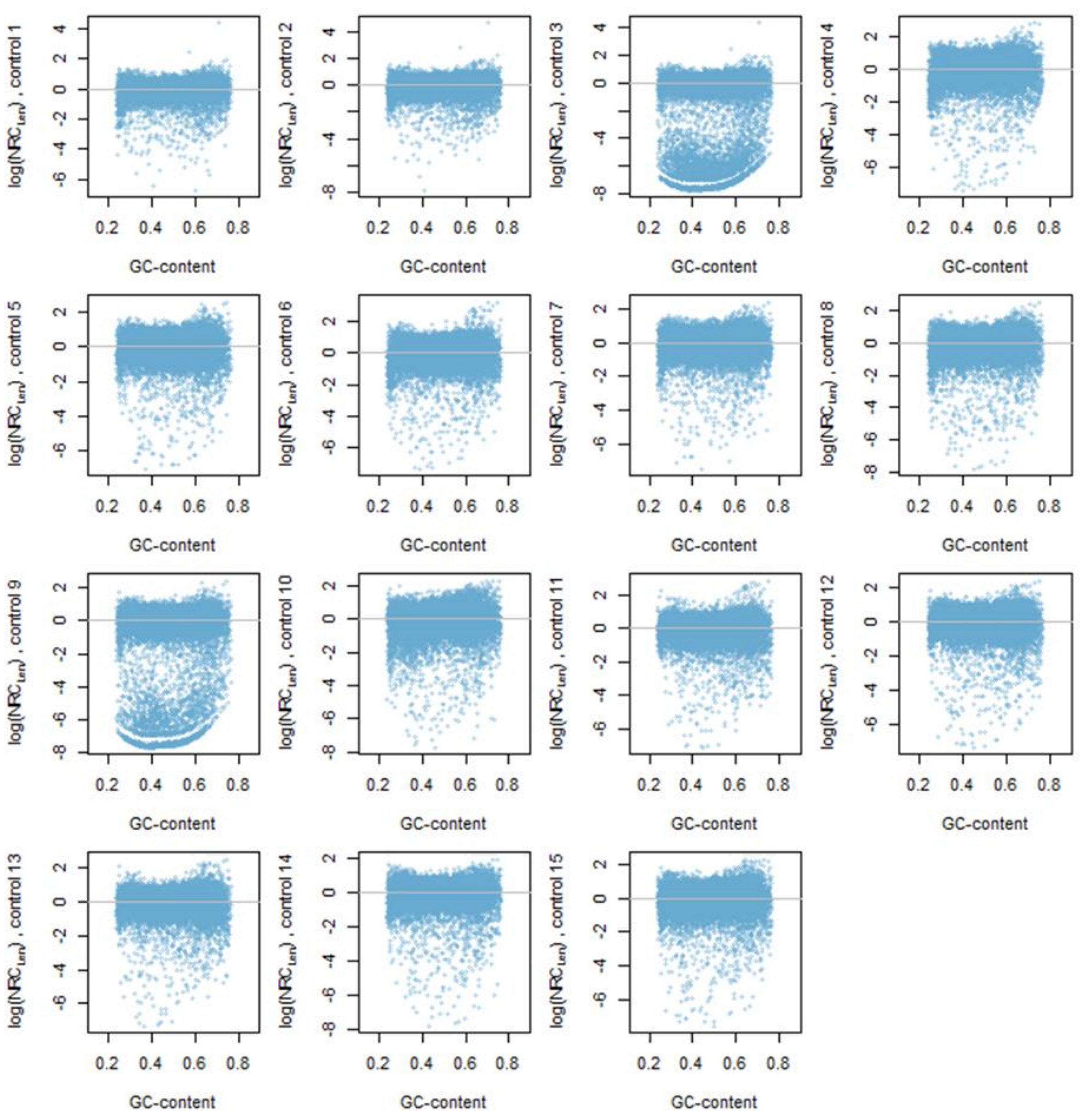

log(NRC_len), control 1
log(NRC_len), control 2
log(NRC_len), control 3
log(NRC_len), control 4
log(NRC_len), control 5
log(NRC_len), control 6
log(NRC_len), control 7
log(NRC_len), control 8
log(NRC_len), control 9
log(NRC_len), control 10
log(NRC_len), control 11
log(NRC_len), control 12
log(NRC_len), control 13
log(NRC_len), control 14
log(NRC_len), control 15
GC-content

D

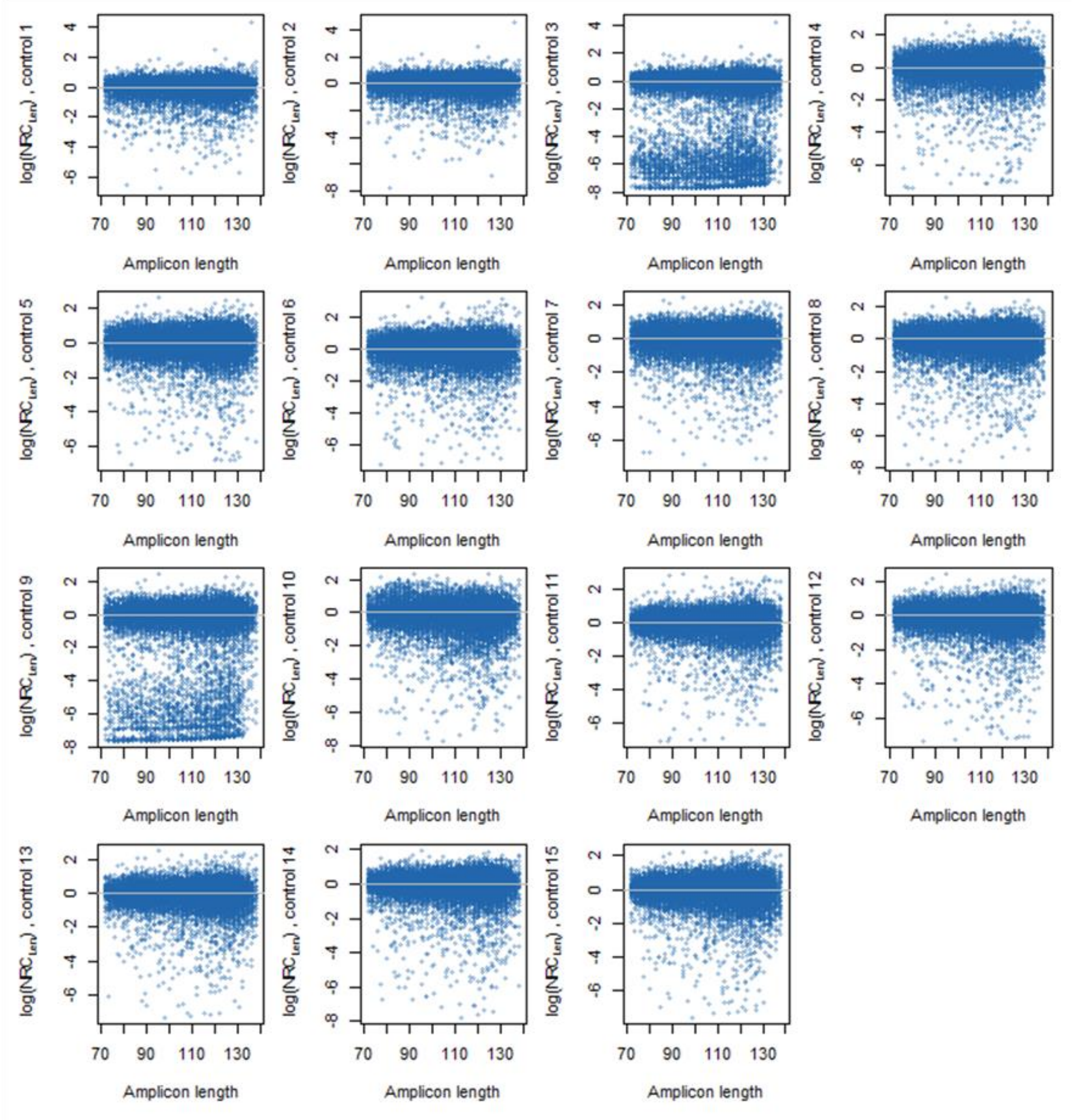

Supplementary Figure 3. Amplicon length bias in the amplicon sequencing data (dataset X)

A-B. Logarithms of read count normalized with regard to library size (log(NRC_{GC})) *versus* amplicon length. **C-D**. Logarithms of read count normalized with regard to amplicon length (log(NRC_{Len})) *versus* GC-content (C) or amplicon length (D).

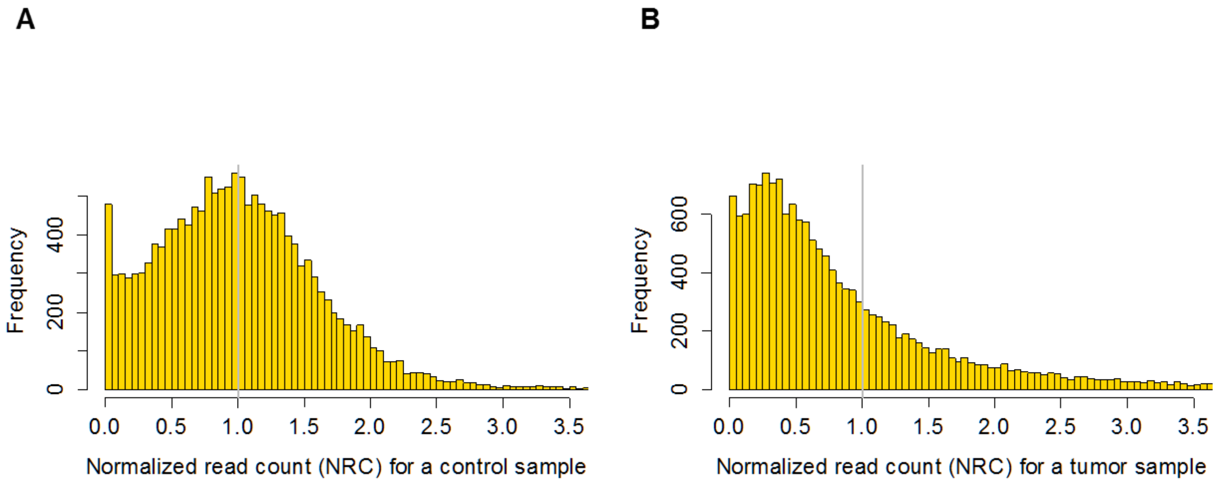

Supplementary Figure 4. Distribution of read counts normalized with regard to library size (NRC_{Lib})

A. Normal control sample X1 (median NRC_{Lib} = 0.96); **B**. Tumor sample B1 (median NRC_{Lib} = 0.61).

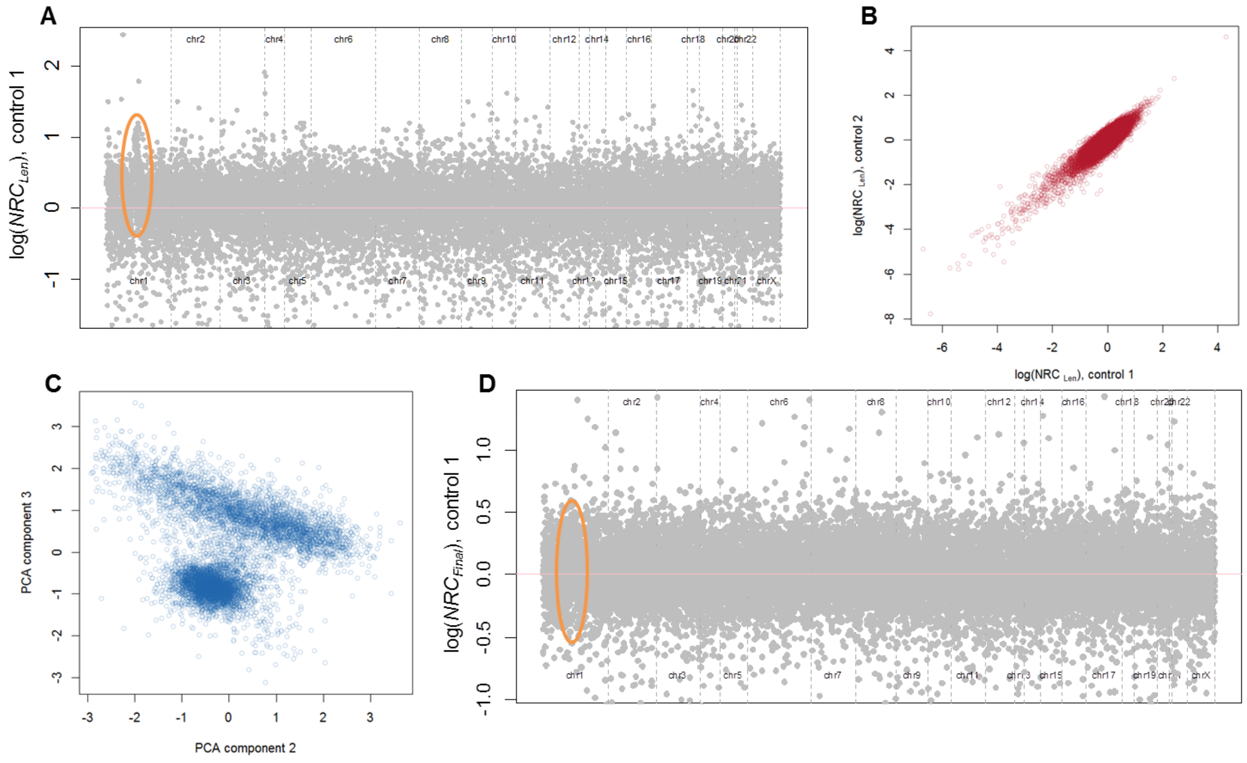

Supplementary Figure 5. Baseline creation

A. Read count profile after the 3 normalization steps (Control sample X1). In orange, a region with extremely high normalized read count values. **B**. Correlation between normalized read count profiles (Control samples X1 and X2). **C**. Second and third components of PCA performed on 15 normalized control samples (dataset X). **D**. Final profile of normalized read count (log(NRC_{Final})) (Control sample X1). The removed bias cannot correspond to the germline copy number changes. First, the baseline is calculated using at least (n+1) diploid control samples, where *n* is the number of principal components to keep. Thus, even if one of the control samples contains a real gain or loss, this event is unlikely to be included into the baseline. Second, the targeted regions in the amplicon sequencing almost always correspond to the exons of genes coding for proteins with important cellular functions. Thus, it is also very unlikely that such genes carry copy number changes in exons at the constitutional level.

A

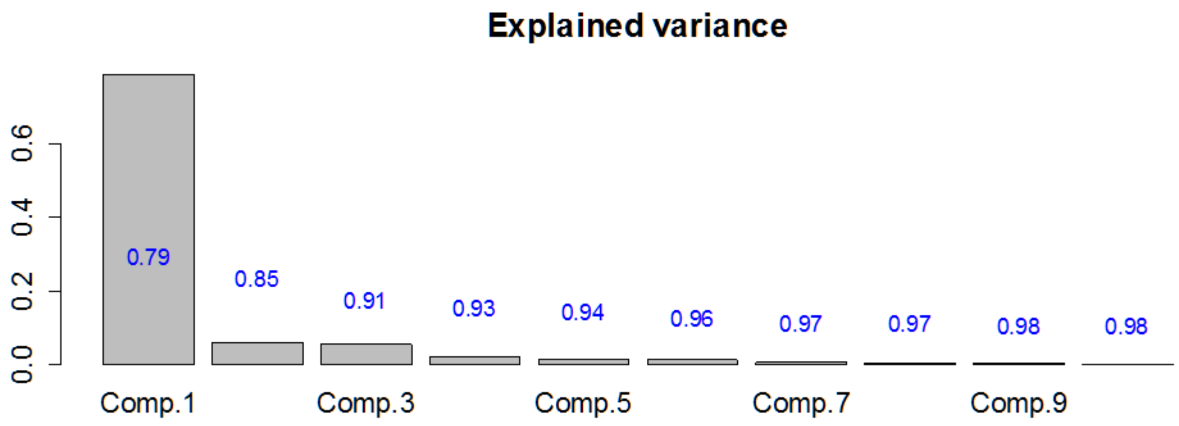

B

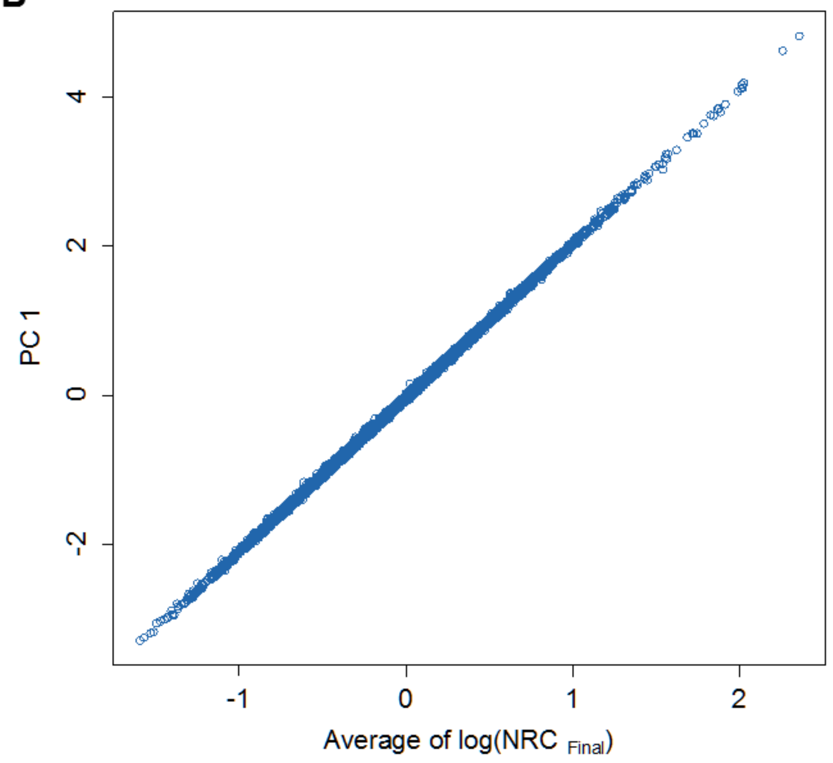

Supplementary Figure 6. Properties of principal components extracted from normalized read counts for control samples (dataset X)

A. Variance explained by first 10 principal components in log(NRC_{Len}) values for 15 controls. The cumulative percent of variance explained by the first principal components is shown in blue. **B.** Correlation between first principal component and average value of log(NRC_{Len}) over 15 controls.

A

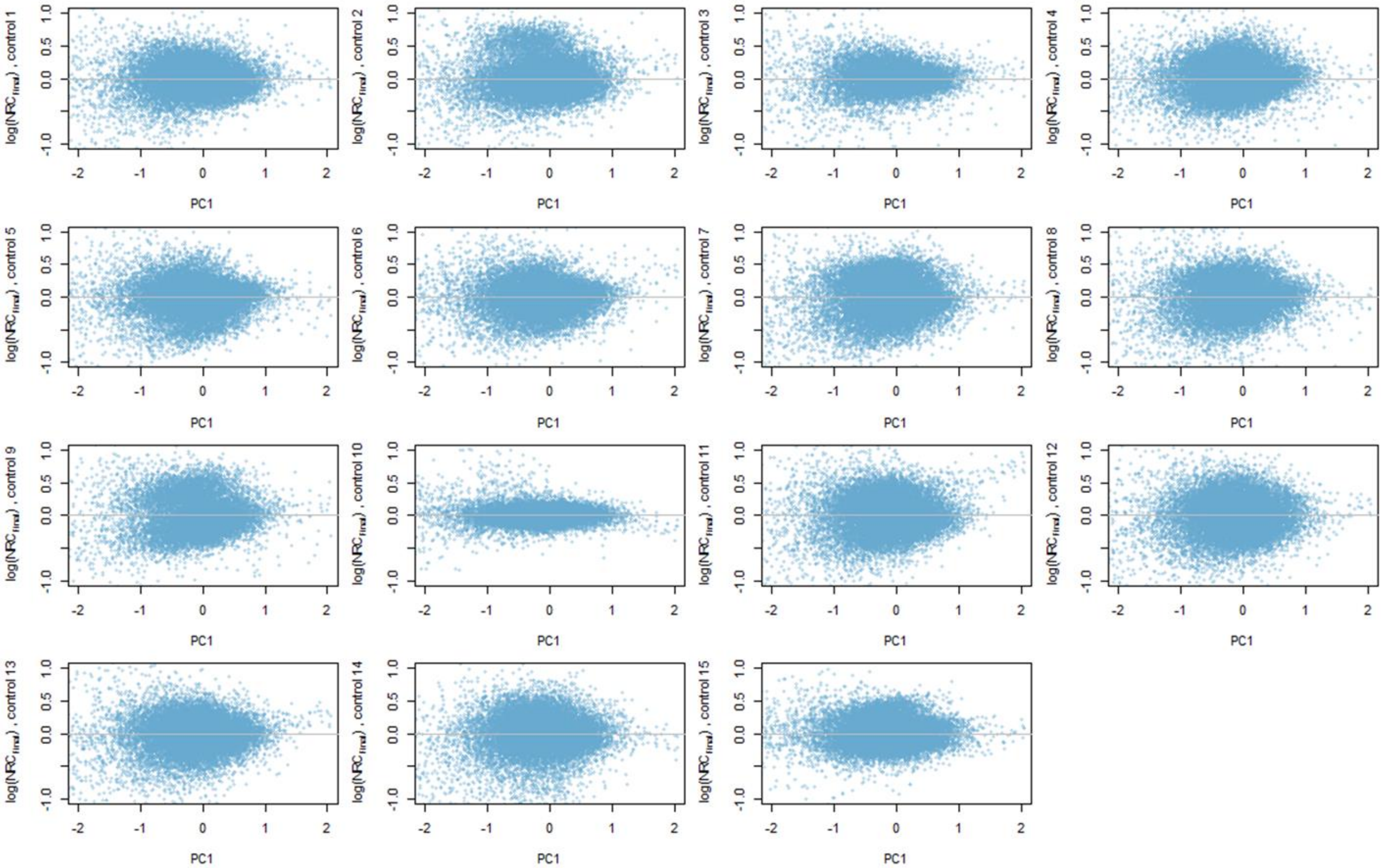
log(NRC_final), control 1
log(NRC_final), control 2
log(NRC_final), control 3
log(NRC_final), control 4
log(NRC_final), control 5
log(NRC_final), control 6
log(NRC_final), control 7
log(NRC_final), control 8
log(NRC_final), control 9
log(NRC_final), control 10
log(NRC_final), control 11
log(NRC_final), control 12
log(NRC_final), control 13
log(NRC_final), control 14
log(NRC_final), control 15
PC1

B

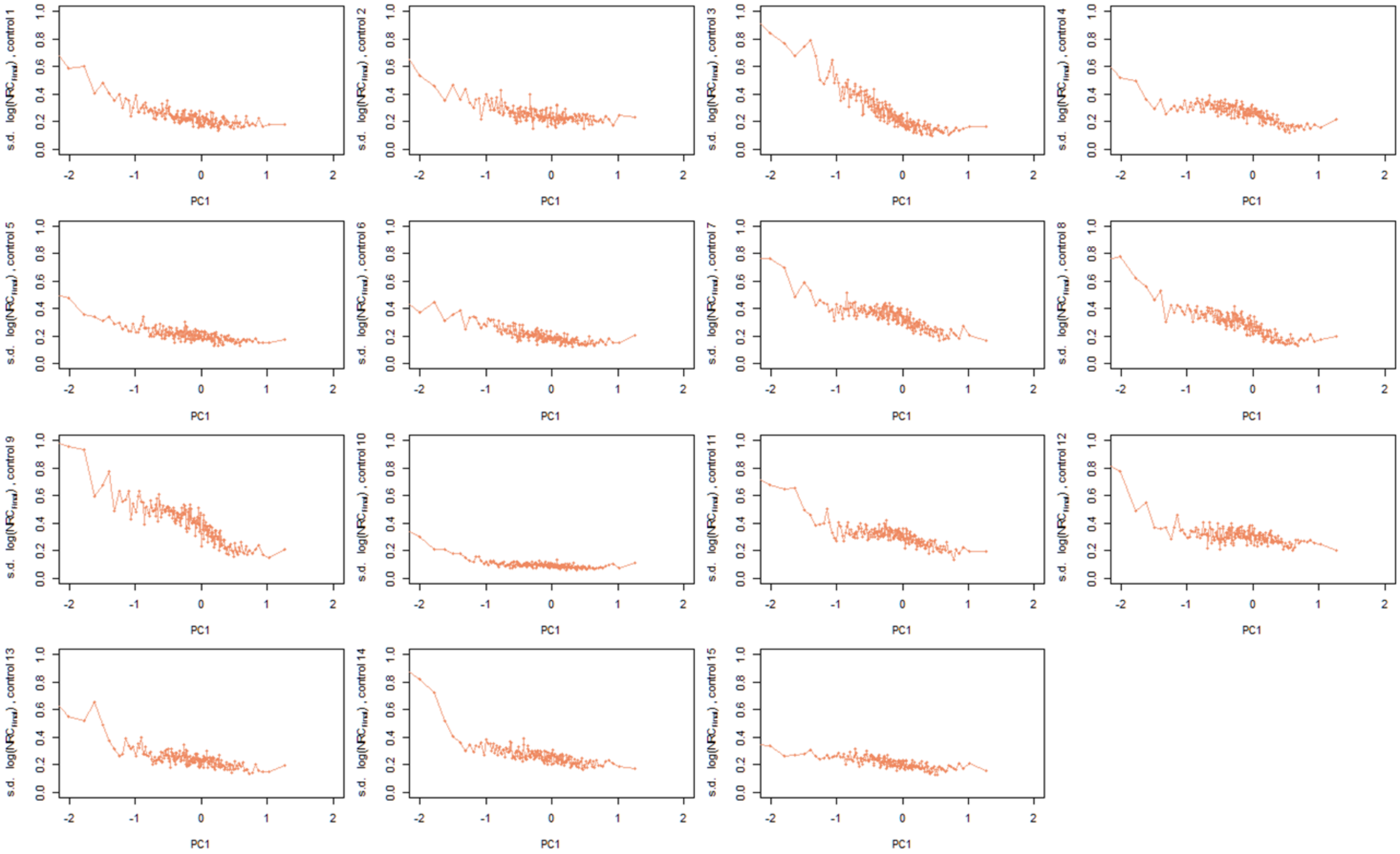

Supplementary Figure 7. Variance in NRC_{final} values depends on first principal component (PC1) values. (**A**) Graphs produced before rescaling for overall variance (dataset X). (**B**) Standard deviation of NRC_{final} values estimated as a function of PC1 (dataset X).

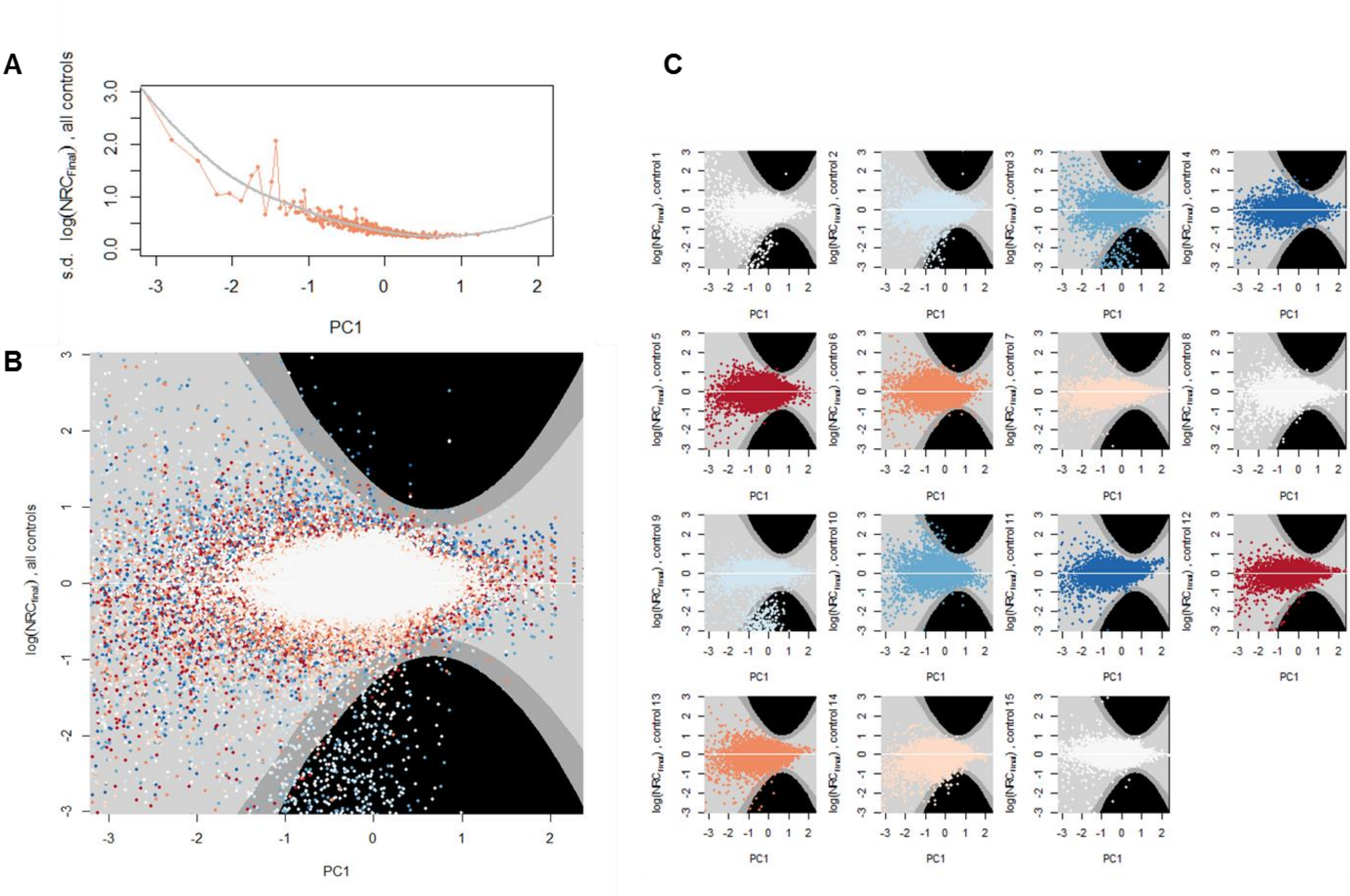

Supplementary Figure 8. LOESS fitting for $\sigma = f(PC1)$

A. Standard deviation of NRC_{final} values estimated as a function of PC1 (dataset X, NRC_{final} values of all control samples merged). LOESS fit shown in grey. **A**. NRC_{final} values (rescaled) for 15 controls (dataset X) in one plot. **B**. Control datasets shown separately. Black = area corresponding to $(-\infty, -4\sigma)$ and $(4\sigma, \infty)$, light gray = area corresponding to $(-3\sigma, 3\sigma)$.

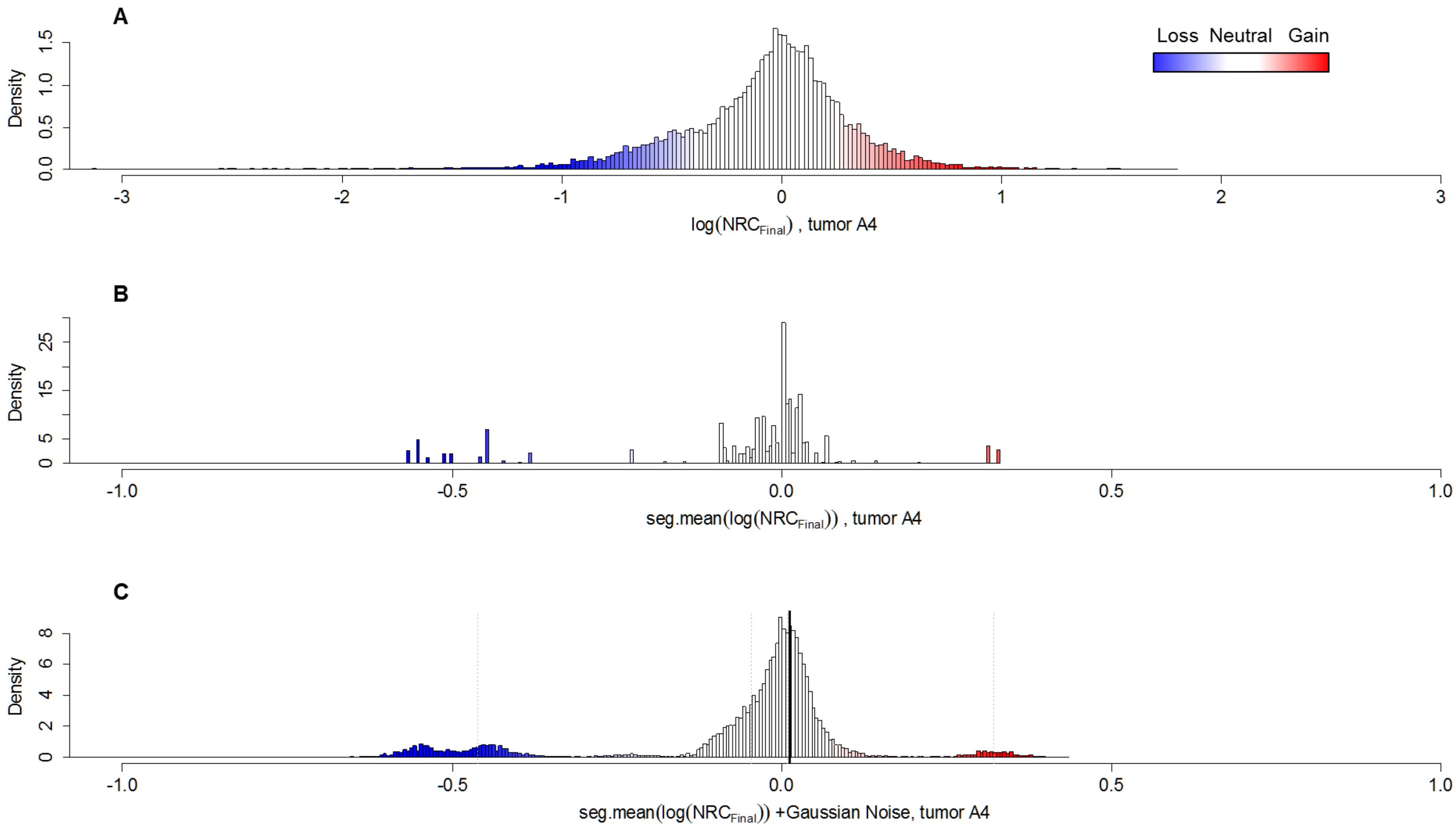

Supplementary Figure 9. Segmentation and clustering approach

A. log(NRC) values. No distinction between gains and losses due to the presence of noise. **B**. Weighted mean values of log(NRC) after segmentation. **C**. Slightly perturbed weighted mean values of log ratios: Random noise $\sim N(0, \sigma^2{}_{seg})$ added, where σ_{seg} is the standard error of the weighted mean of the corresponding segment. Putative gains and losses corresponding to high densities to left and right of zero (black solid line). Centers of clusters shown by vertical dashed gray lines.

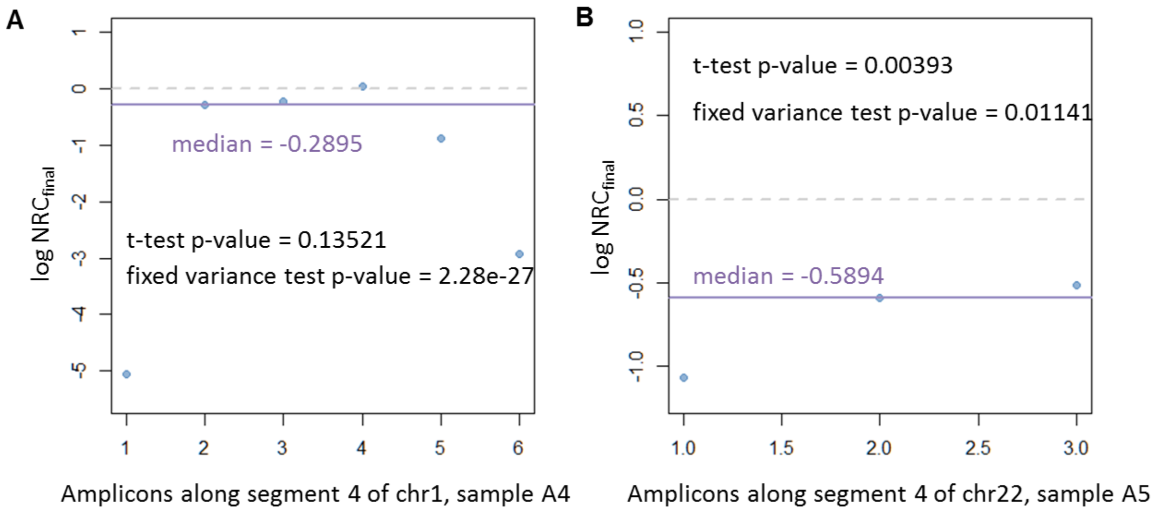

Supplementary figure 10. ONCOCNV uses two statistical tests to prevent the prediction of false positive copy number alterations: the t-test and the fixed variance test. ONCOCNV keeps only those candidate CNAs for which the fixed variance test and t-test p-values are lower than 0.01. (**A**) A situation when the fixed variance test produces a significant p-value while the t-test does not (Sample A4, chr1, segment 4 from 42525784 to 42526792 bp, annotated as copy-neutral by the array CGH analysis). (**B**) A situation when the t-test produces a significant p-value while the fixed variance test does not (Sample A5, chr22, segment 4 from 65321270 to 65330546 bp, annotated as copy-neutral by the array CGH analysis). These examples demonstrate that using a threshold on the p-values of both tests allows ONCOCNV to discard false positive predictions. Indeed, the t-test allows us to discard short segments with high overall variance (left panel) that contains several amplicon regions with extremely high or low NRCs (this situation results in a significant p-value of the fixed variance test), while the fixed variance test allows us to discard short regions that, by chance, contain only very close NRC values, all positive or all negative, with relatively small absolute values (this situation results in a significant p-value of the t-test).

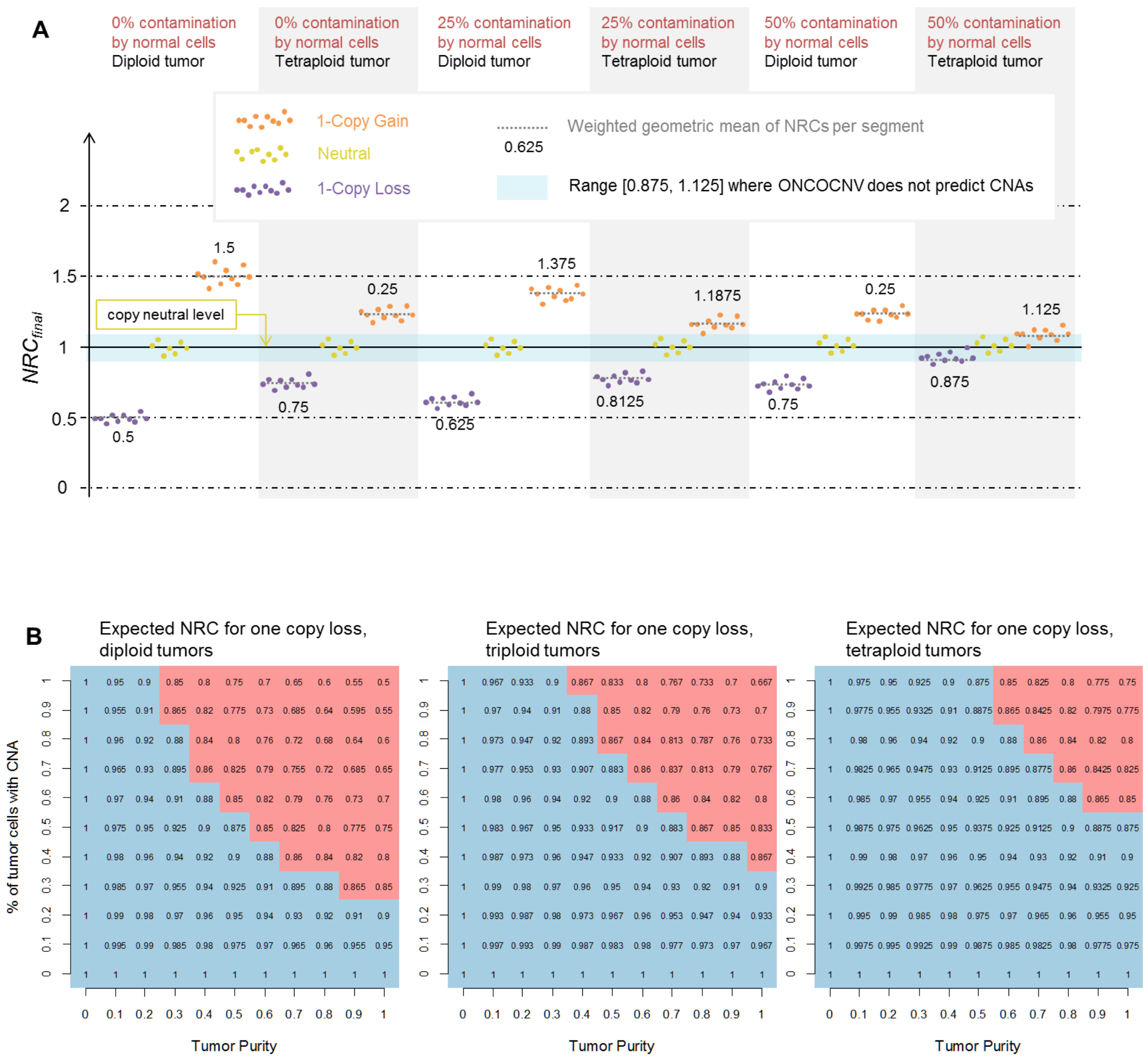

Supplementary figure 11. Theoretical sensitivity of CNA detection by ONCOCNV

A. "Shrinkage" of NRC values towards 1 with higher tumor ploidy and higher contamination by normal cells does not allow for the correct CNA detection in highly contaminated or/and hyperploid tumors: loss of one copy (purple), gain of one copy (orange), copy neutral region (yellow). ONCOCNV filters out all candidate CNAs whose weighted geometric mean of NRCs falls within the range [0.875, 1.125] (light blue). **B**. This filter is likely to remove all CNAs present in less than 25% of cells of diploid tumors, in less than 33% of cells in triploid tumors and in less than 50% of cells of tetraploid tumors. Combination of tumor purity and clonality allowing a one-copy CNA to pass the filter: red (passed), blue (rejected).

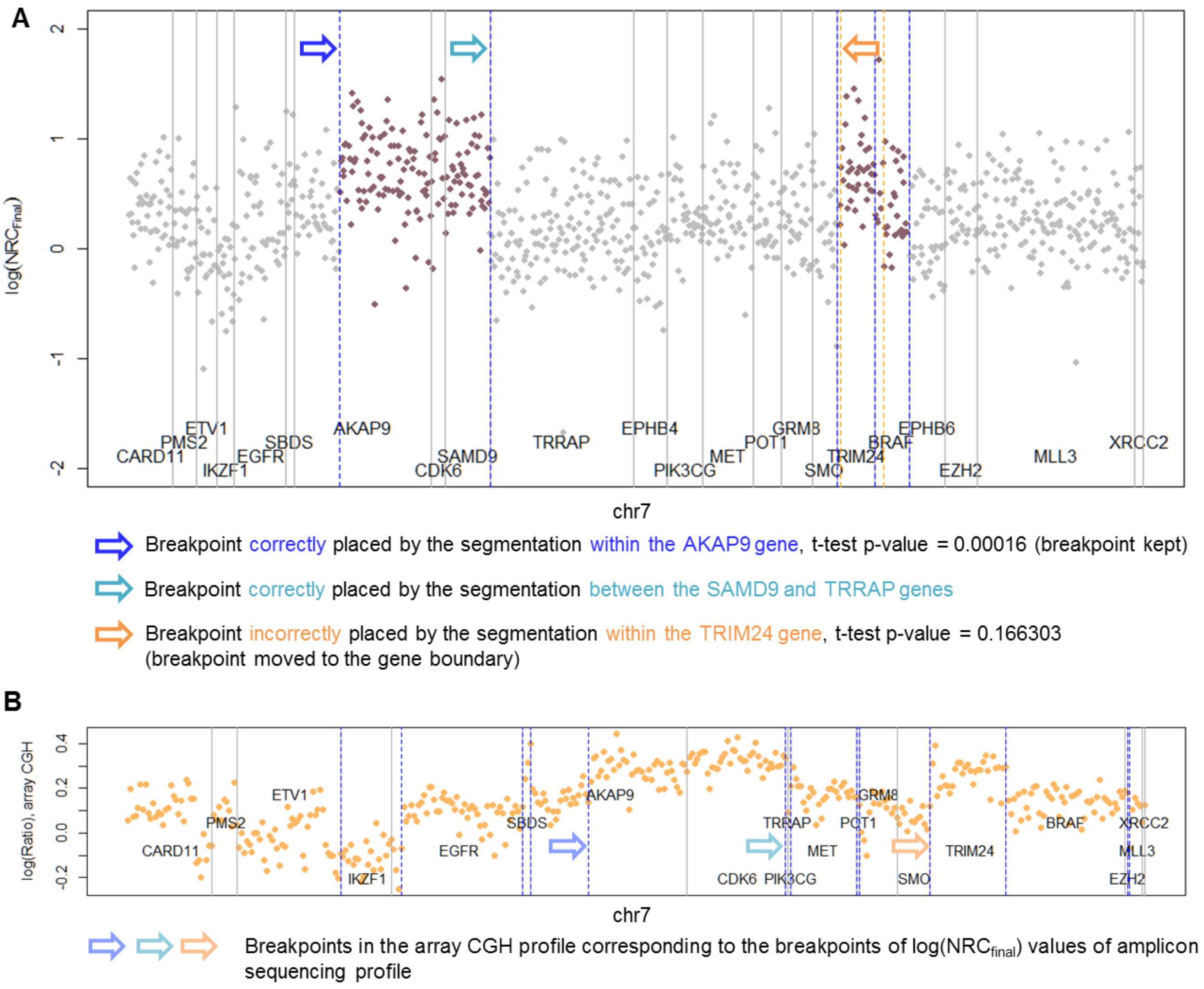

Supplementary Figure 12. Segmentation in a gene-unaware way can misplace breakpoints

A. Circular binary segmentation of log NRC values (sample A1, chr 7). Dot color corresponds to the final copy number status: gain of more than one copy (brown), other (grey); genes delimited by gray solid lines; final breakpoints (blue); misplaced breakpoints (orange). **B**. Log (Ratio) values from the targeted genes (sample A1, chr7). Segmentation breakpoints (blue).

Sample A1

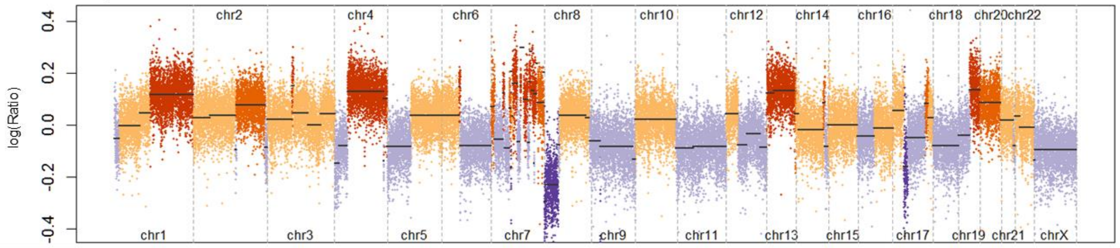

Sample A2

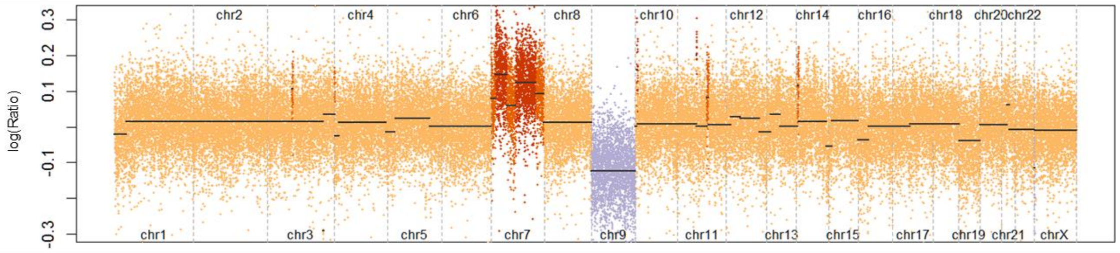

Sample A3

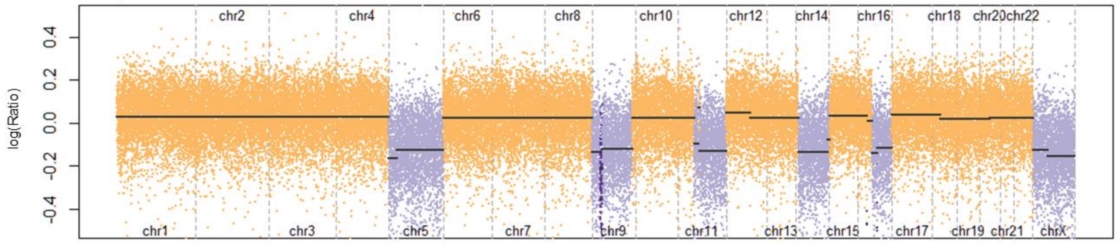

Sample A4

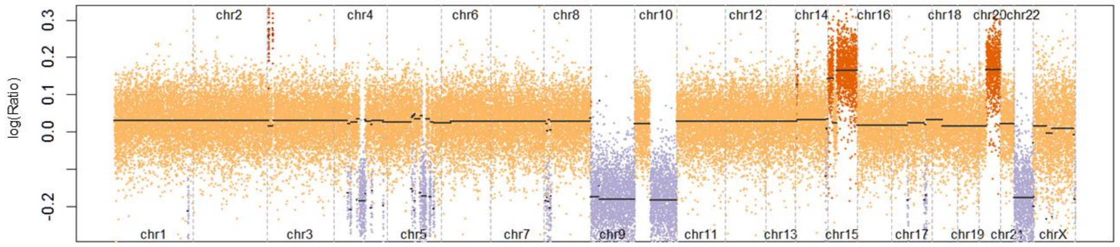

Sample A5

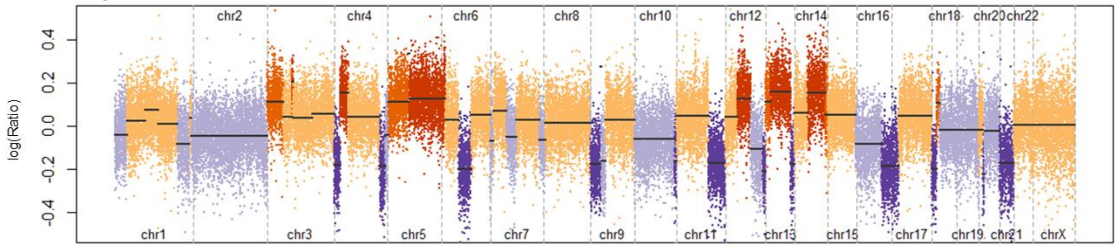

Sample A6

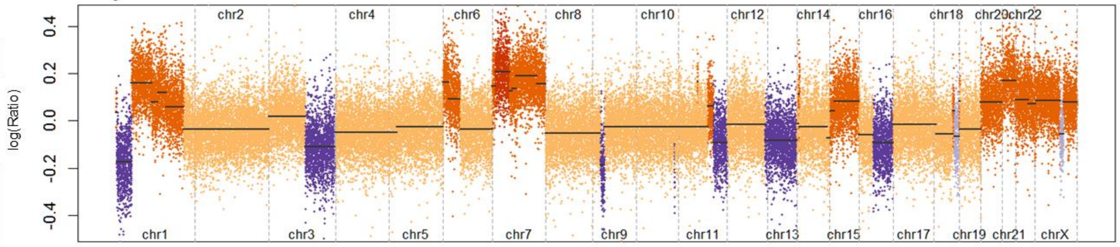

Sample A7

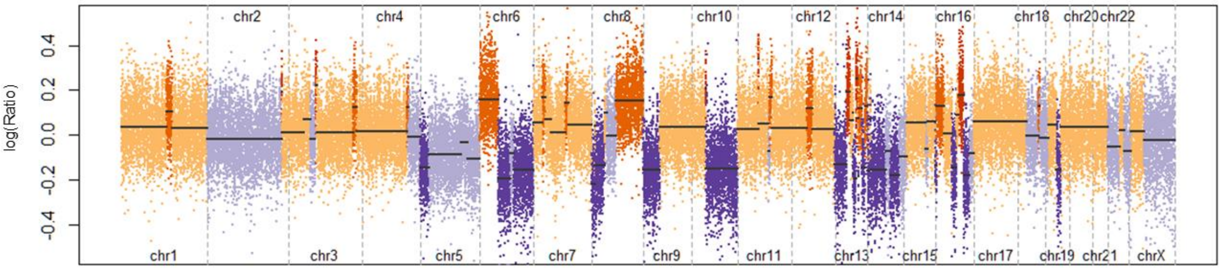

Sample A8

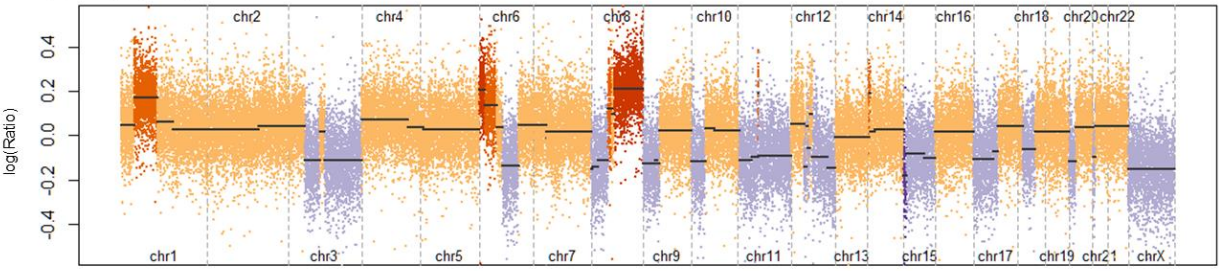

Supplementary Figure 13. Array CGH copy number profile for samples A1-A8, segmented using cghseg (13)

Purple=loss, orange=gain. X-axis corresponds to probe indexes.

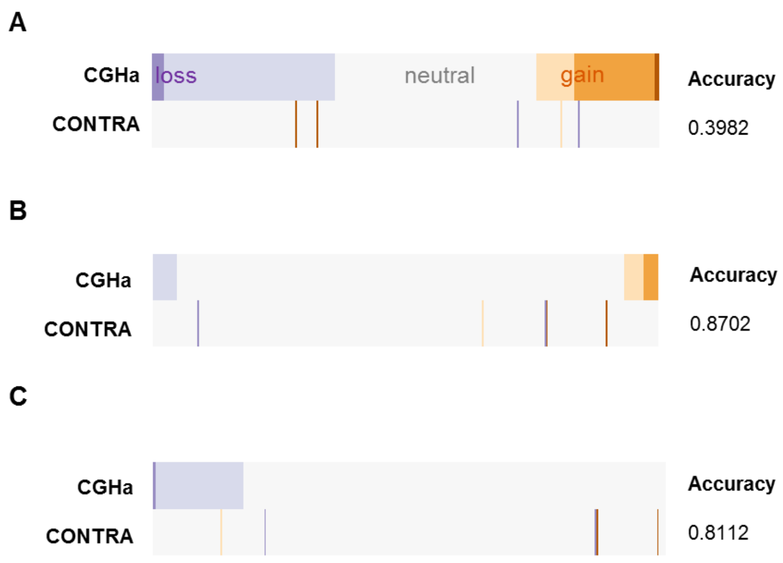

Supplementary Figure 14. Agreement between CNVs predicted from CGH arrays and amplicon sequencing by CONTRA software

White=neutral, purple=loss, orange=gain. **A**. Sample A1. **B**. Sample A2. **C**. Sample A3. Baseline for CONTRA created using 13 normal control samples. Control samples 3 and 9 excluded from analysis because of poor amplification of ≥25% of amplicon regions in these samples.

Parameters used to run CONTRA: Baseline was created using the default parameters and 13 diploid controls. We excluded controls 3 and 9 since they showed a high number of targeted regions with low amplification rate. The script “contra.py” was run with default parameters; the tag “--largeDeletion” was specified to allow for the detection of large CNVs using circular binary segmentation of the copy number profile.

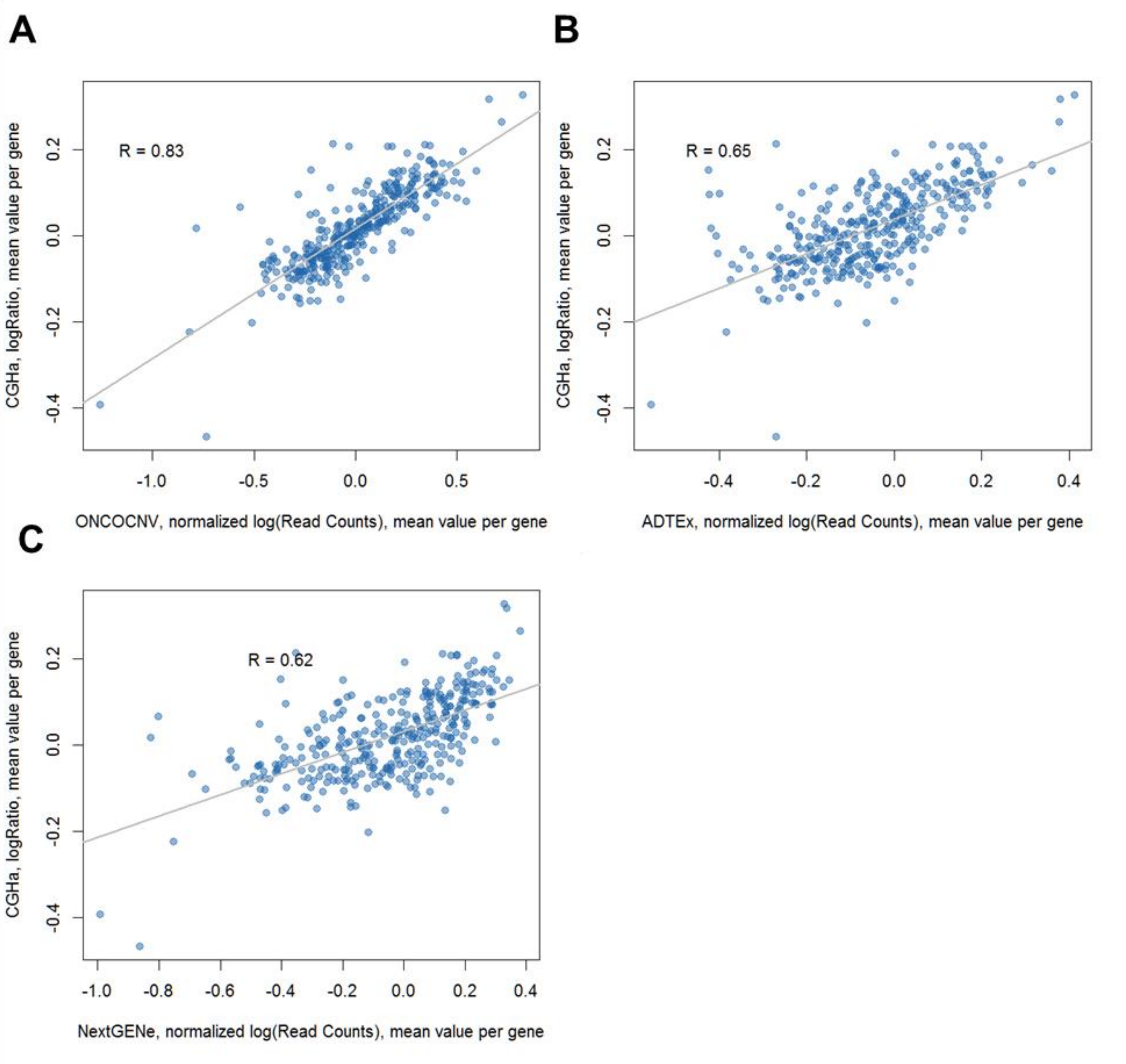

Supplementary Figure 15. Correlation between log ratio values of array CGH and read counts normalized by (**A**) ONCOCNV, (**B**) ADTEx and (**C**) NextGENe (Sample A1). Mean value per gene is shown.

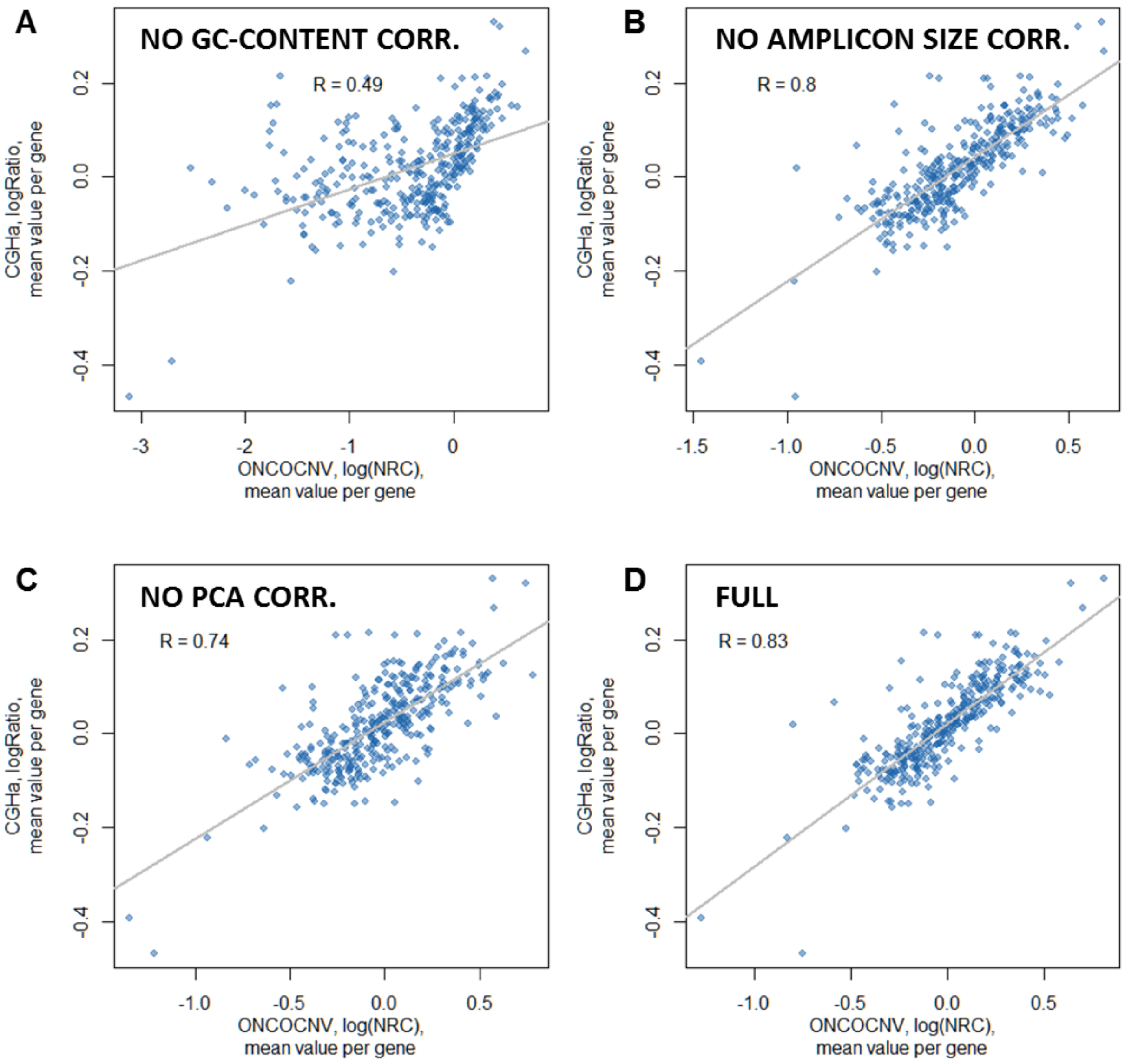

	Pearson R	Spearman rho
No GC	0.49	0.54
No A.Lengths	0.80	0.81
No PCA	0.74	0.72
**Full**	**0.83**	**0.84**
ADTEx	0.65	0.65
NextGene	0.62	0.62

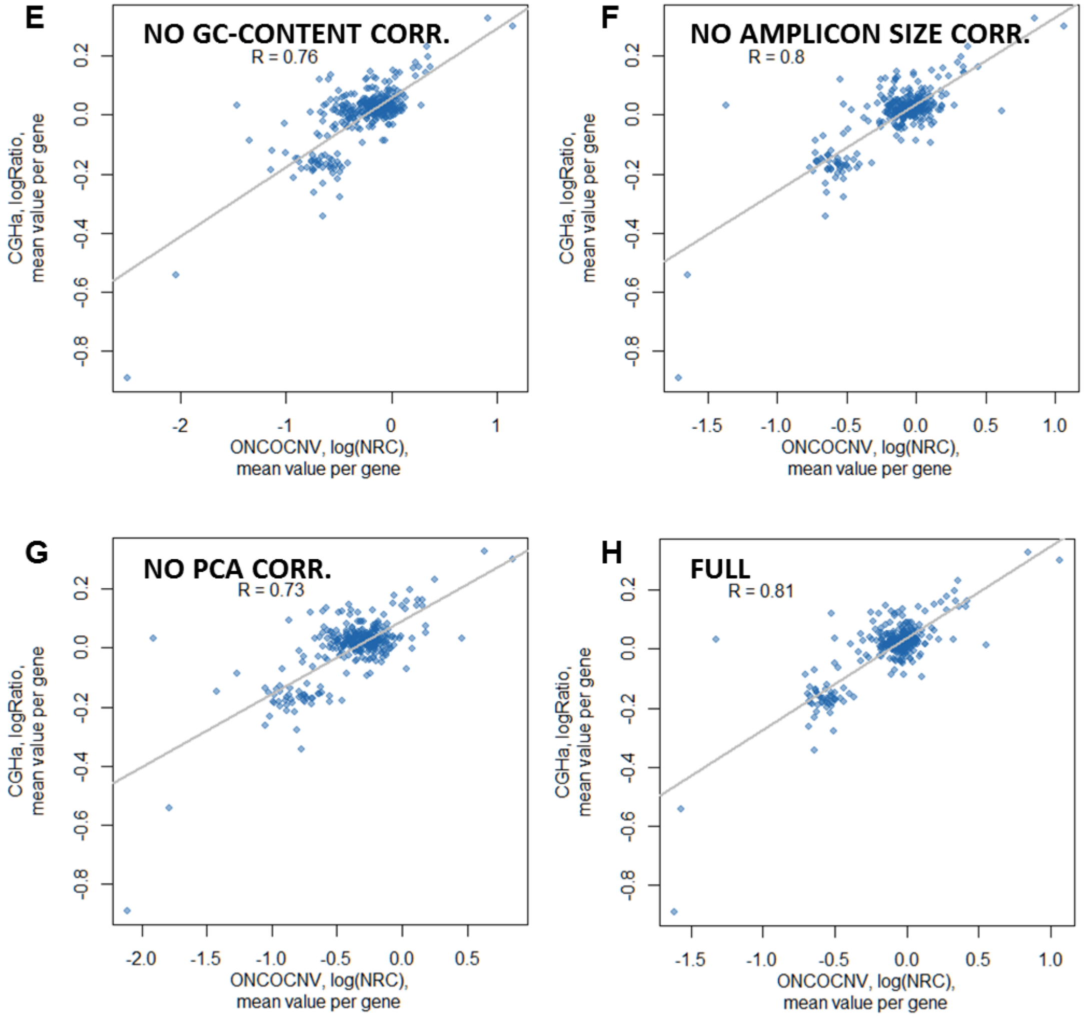

	Pearson R	Spearman rho
No GC	0.76	0.54
No A.Lengths	0.80	0.52
No PCA	0.73	0.44
**Full**	**0.81**	**0.51**
ADTEx	0.64	0.39
NextGene	0.63	0.43

Supplementary figure 16. Relative importance of different normalization steps in ONCOCNV

The importance of each normalization step was demonstrated on two tumor samples with high proportion of gains and losses in the genome (Sample A1: A-D, Sample A4: E-H). The correlation between normalized read counts and array CGH log ratio values (mean value per gene) served as a measure of the final normalization quality. (**A, E**) Normalization includes all normalization steps except GC-content correction; (**B, F**) Normalizationincludes all normalization steps except the correction for amplicon lengths; (**C, G**) Normalization includes all normalization steps except the correction for the first components of PCA, i.e. technology-based bias; (**D, H**) Normalization includes all normalization steps.

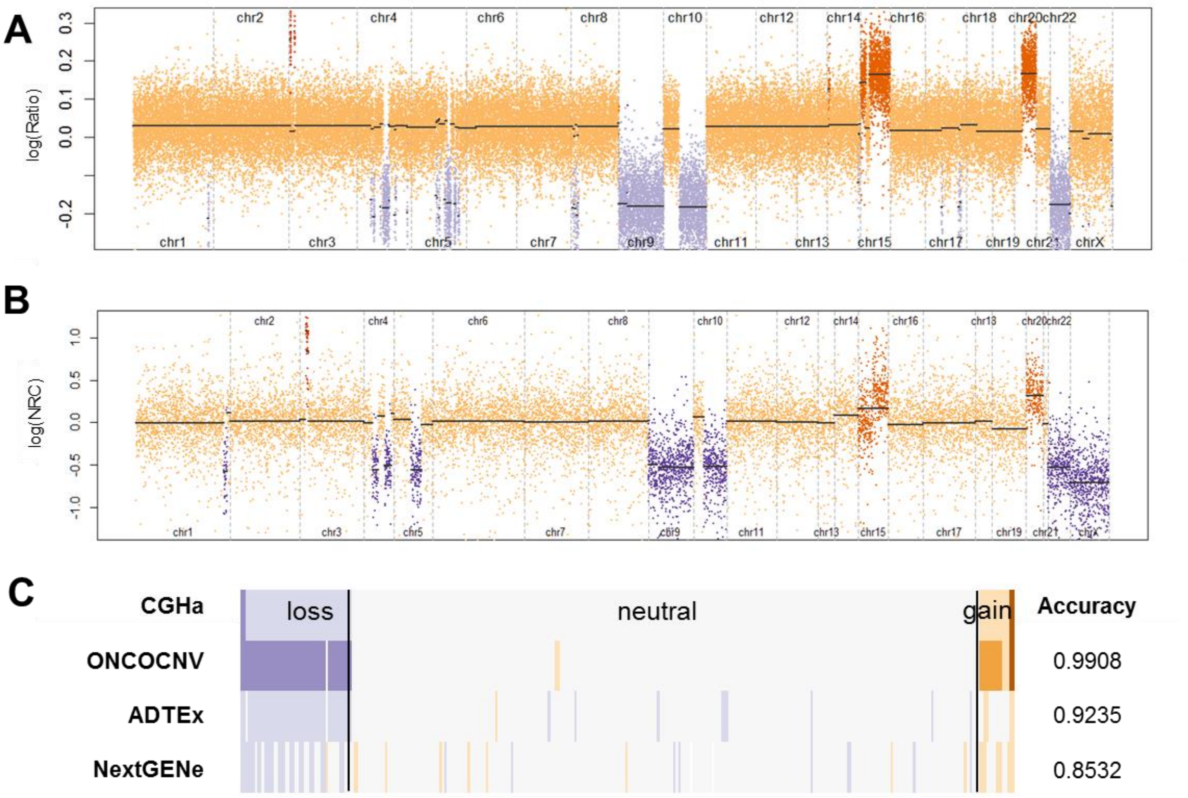

Supplementary Figure 17. Comparison of CNVs called by ONCOCNV, ADTEx and NextGENe with predictions based on array CGH data for a low complexity profile (Sample A4)

A. CGH profile for sample A4, segmented using cghseg (13): purple (loss), orange (gain). X-axis corresponds to probe indexes. **B**. Copy number profile calculated by ONCODNA. X-axis corresponds to amplicon indexes. **C**. Agreement between CNVs predicted from array CGHs and amplicon sequencing (chromosome X is excluded). Each vertical bar denotes a gene copy number status: white (neutral), purple (loss), orange (gain). $Accuracy = \frac{\#True\ Predictions}{\#All\ predictions}$, where each prediction corresponds to a gene copy number status (gain, neutral or loss).

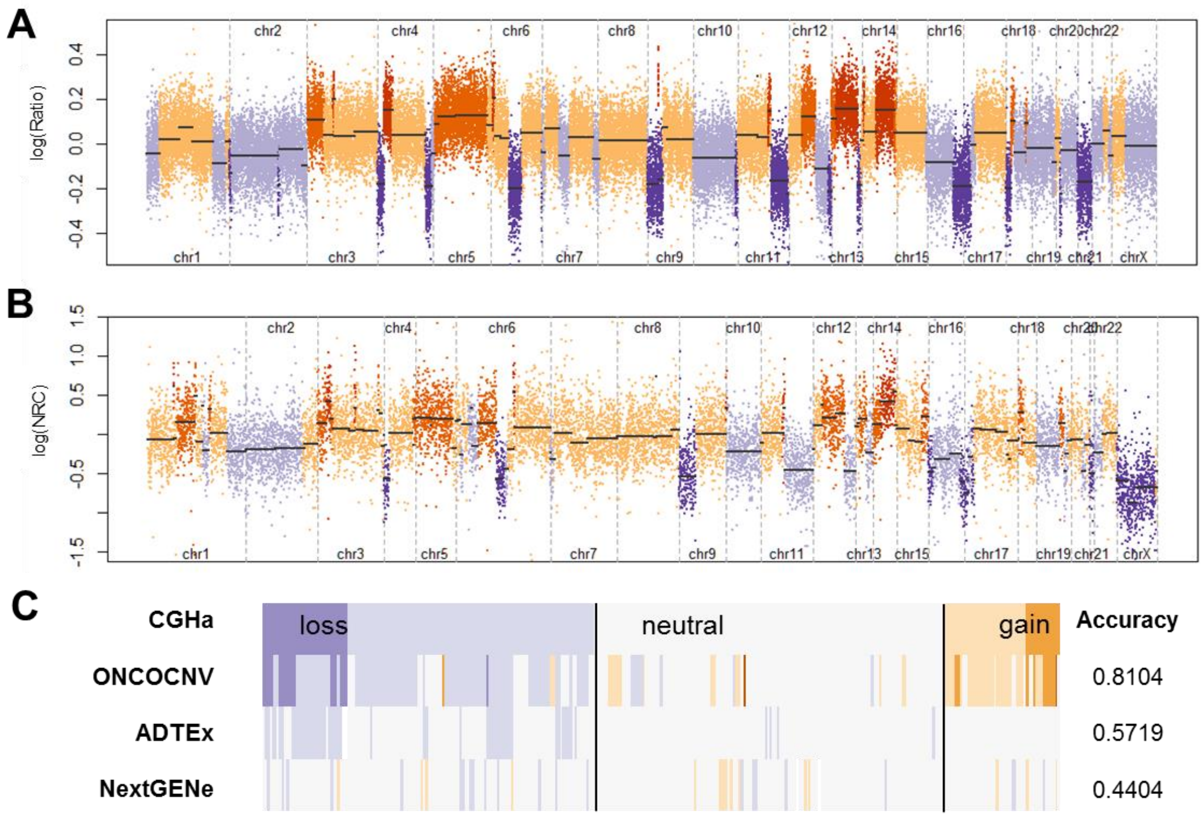

Supplementary Figure 18. Comparison of CNVs called by ONCOCNV, ADTEx and NextGENe with predictions based on array CGH data for a high complexity profile (Sample A5)

A. CGH profile for sample A5, segmented using cghseg[17]: purple (loss), orange (gain). X-axis corresponds to probe indexes. **B**. Copy number profile calculated by ONCODNA. X-axis corresponds to amplicon indexes. **C**. Agreement between CNVs predicted from array CGHs and amplicon sequencing (chromosome X is excluded). Each vertical bar denotes a gene copy number status: white (neutral), purple (loss), orange (gain). $Accuracy = \frac{\#True\ Predictions}{\#All\ predictions}$, where each prediction corresponds to a gene copy number status (gain, neutral or loss).

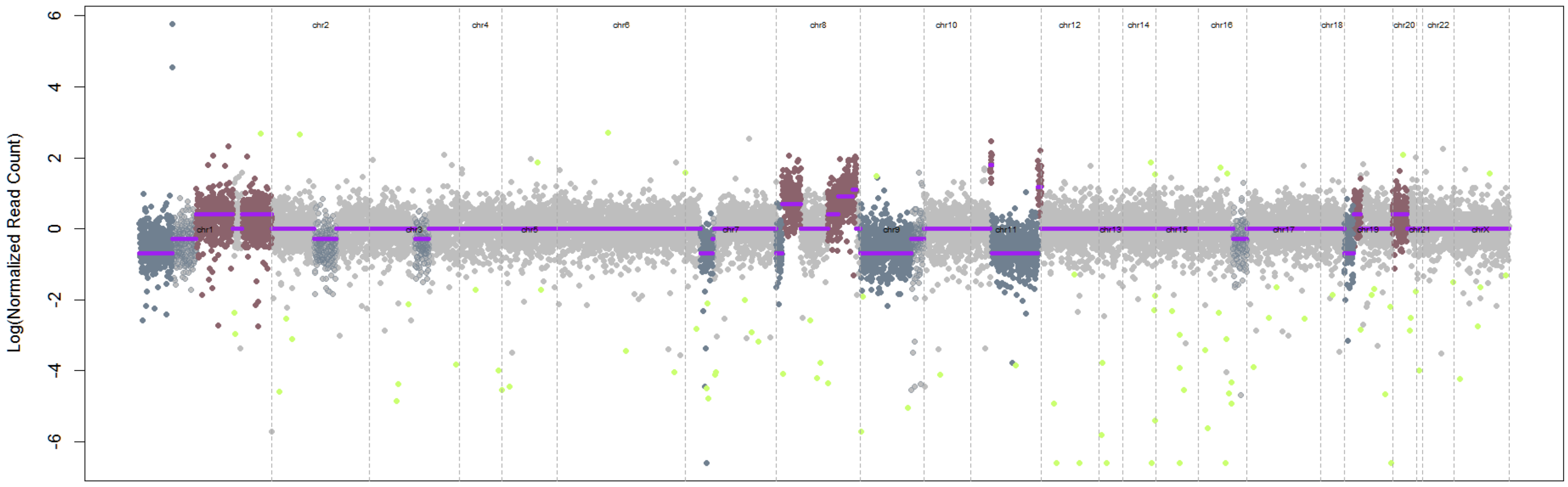

Supplementary Figure 19. Visualization of normalized read count profile for ErbB2-positive sample showing no ERBB2-gain when analyzed by ONCOCNV (Sample C27)

Color corresponds to predicted copy number status: gray=normal, brown=gain, blue=loss.

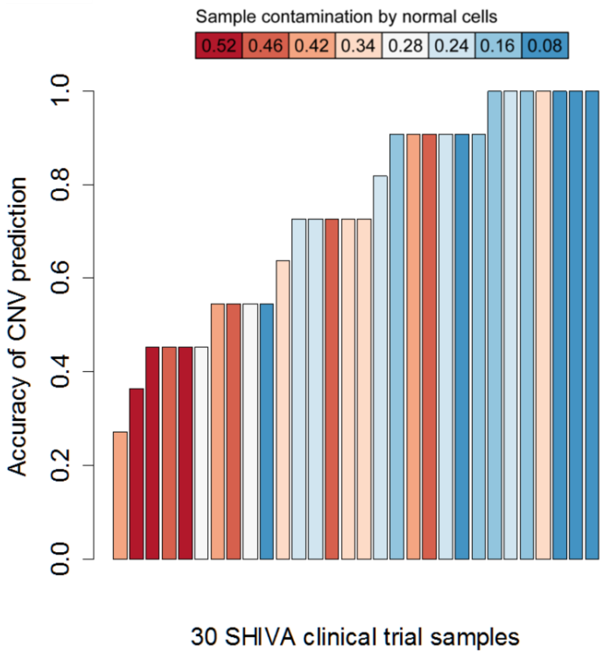

Supplementary figure 20. Absolute CNV detection accuracy in 30 samples from the SHIVA clinical trial (dataset C).

Supplementary Tables

Supplementary Table 1. Correlation between log ratio values of arrays CGH and read counts normalized by different methods

	Spearman Rho/Pearson R							
**Method**	**Sample A1**	**Sample A2**	**Sample A3**	**Sample A4**	**Sample A5**	**Sample A6**	**Sample A7**	**Sample A8**
ONCOCNV	0.84/0.83	0.59/0.61	0.61/0.63	0.51/0.81	0.81/0.74	0.70/0.69	0.80/0.78	0.68/0.68
ADTEx, single control	0.66/0.66	0.51/0.50	0.41/0.40	0.26/0.48	0.58/0.54	0.57/0.53	0.71/0.70	0.52/0.48
ADTEx, merged controls	0.65/0.66	0.56/0.57	0.43/0.43	0.25/0.50	0.59/0.53	0.58/0.52	0.70/0.69	0.51/0.47
ADTEx, single control, no duplicates	0.65/0.65	0.54/0.52	0.42/0.42	0.39/0.64	0.65/0.66	0.60/0.62	0.76/0.74	0.58/0.56
ADTEx, merged controls, no duplicates	0.63/0.65	0.50/0.49	0.38/0.38	0.30/0.54	0.61/0.60	0.52/0.49	0.67/0.66	0.49/0.46
NextGene	0.62/0.62	0.58/0.58	0.38/0.38	0.43/0.63	0.33/0.30	0.59/0.58	0.29/0.27	0.15/0.14

Supplementary Table 2. Accuracy of CNV prediction. True positive rate (TP), true negative rate (TN), false positive rate (FP), false negative rate (FN).

**Sample A1**			
	**ONCOCNV**	**ADTEx**	**NextGENe**
TP	0.8627	0.4167	0.3873
TN	0.9259	0.8815	0.7333
FP	0.0741	0.1037	0.2593
FN	0.1176	0.5735	0.5882
Accuracy	0.8879	0.6018	0.5251

**Sample A2**			
	**ONCOCNV**	**ADTEx**	**NextGENe**
TP	0.8205	0.4359	0.6154
TN	1.0000	0.8800	0.7133
FP	0.0000	0.1133	0.2800
FN	0.1795	0.5641	0.3846
Accuracy	0.9794	0.8289	0.7021

**Sample A3**			
	**ONCOCNV**	**ADTEx**	**NextGENe**
TP	0.9848	0.1061	0.5152
TN	0.9927	0.6960	0.7106
FP	0.0073	0.2967	0.2821
FN	0.0152	0.8788	0.4848
Accuracy	0.9912	0.5811	0.6726

**Sample A4**			
	**ONCOCNV**	**ADTEx**	**NextGENe**
TP	0.9839	0.7903	0.5484
TN	0.9925	0.9547	0.9245
FP	0.0075	0.0377	0.0679
FN	0.0161	0.2097	0.4355
Accuracy	0.9908	0.9235	0.8532

**Sample A5**			
	**ONCOCNV**	**ADTEx**	**NextGENe**
TP	0.7935	0.2609	0.0924
TN	0.8322	0.9720	0.8881
FP	0.1678	0.0280	0.0979
FN	0.1902	0.7283	0.8696
Accuracy	0.8104	0.5719	0.4404

Sample A6			
	ONCOCNV	ADTEx	NextGENe
TP	0.8079	0.0728	0.2583
TN	0.9468	0.9468	0.8830
FP	0.0532	0.0532	0.1064
FN	0.1921	0.9007	0.7020
Accuracy	0.8850	0.5575	0.6047

Sample A7			
	ONCOCNV	ADTEx	NextGENe
TP	0.5951	0.3865	0.0552
TN	0.8902	0.9451	0.9207
FP	0.1098	0.0427	0.0793
FN	0.3865	0.6074	0.8712
Accuracy	0.7431	0.6667	0.4893

Sample A8			
	ONCOCNV	ADTEx	NextGENe
TP	0.8739	0.2605	0.1597
TN	0.8818	0.9136	0.8227
FP	0.1182	0.0773	0.1773
FN	0.1176	0.7395	0.7899
Accuracy	0.8791	0.6844	0.5900
